# Spectroscopic and Computational Observation of Glutamine Tautomerization in the Blue Light Sensing Using Flavin Domain Photoreaction

**DOI:** 10.1021/jacs.2c10621

**Published:** 2023-01-06

**Authors:** Yusaku Hontani, Jennifer Mehlhorn, Tatiana Domratcheva, Sebastian Beck, Miroslav Kloz, Peter Hegemann, Tilo Mathes, John T. M. Kennis

**Affiliations:** † Department of Physics and Astronomy, Vrije Universiteit Amsterdam, 1081 HV Amsterdam, De Boelelaan, The Netherlands; ‡ Institut für Biologie, Experimentelle Biophysik, Humboldt Universität zu Berlin, Invalidenstrasse 42, D-10115 Berlin, Germany; § Department of Biomolecular Mechanisms, Max Planck Institute for Medical Research, 69120 Heidelberg, Germany; ∥ Department of Chemistry, Lomonosov Moscow State University, 119991 Moscow, Russia; ⊥ Department of Chemistry, Humboldt-Universität zu Berlin, Brook-Taylor-Str. 2, 12489 Berlin, Germany; # Institute of Physics, ELI-Beamlines, Na Slovance 2, 182 21 Praha 8, Czech Republic

## Abstract

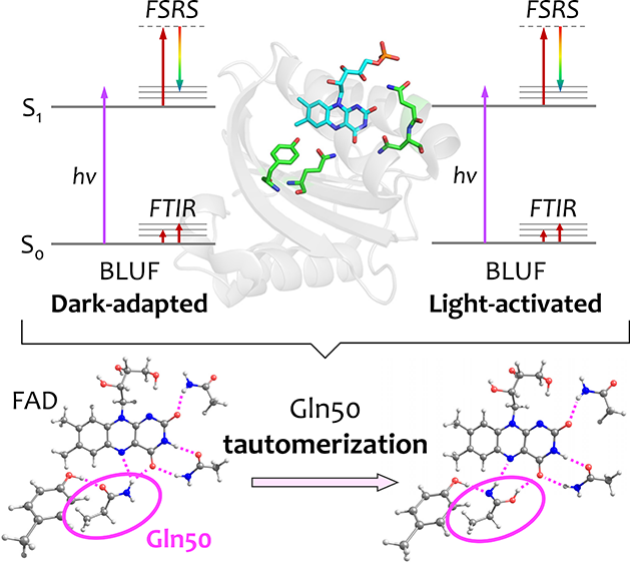

Blue light sensing using flavin (BLUF) domains constitute a family of flavin-binding photoreceptors of bacteria and eukaryotic algae. BLUF photoactivation proceeds *via* a light-driven hydrogen-bond switch among flavin adenine dinucleotide (FAD) and glutamine and tyrosine side chains, whereby FAD undergoes electron and proton transfer with tyrosine and is subsequently re-oxidized by a hydrogen back-shuttle in picoseconds, constituting an important model system to understand proton-coupled electron transfer in biology. The specific structure of the hydrogen-bond patterns and the prevalence of glutamine tautomeric states in dark-adapted (DA) and light-activated (LA) states have remained controversial. Here, we present a combined femtosecond stimulated Raman spectroscopy (FSRS), computational chemistry, and site-selective isotope labeling Fourier-transform infrared spectroscopy (FTIR) study of the Slr1694 BLUF domain. FSRS showed distinct vibrational bands from the FADS_1_ singlet excited state. We observed small but significant shifts in the excited-state vibrational frequency patterns of the DA and LA states, indicating that these frequencies constitute a sensitive probe for the hydrogen-bond arrangement around FAD. Excited-state model calculations utilizing four different realizations of hydrogen bond patterns and glutamine tautomeric states were consistent with a BLUF reaction model that involved glutamine tautomerization to imidic acid, accompanied by a rotation of its side chain. A combined FTIR and double-isotope labeling study, with ^13^C labeling of FAD and ^15^N labeling of glutamine, identified the glutamine imidic acid C=N stretch vibration in the LA state and the Gln C=O in the DA state. Hence, our study provides support for glutamine tautomerization and side-chain rotation in the BLUF photoreaction.

## Introduction

Blue-light using flavin (BLUF) photoreceptors are involved in various functions such as photosynthetic gene regulation,^1−3^ phototaxis,^4^ and enzyme photoregulation.^5,6^ They are of great interest for optogenetic applications^6−10^ and constitute an important model system for proton-coupled electron transfer (PCET) in biology.^11−15^ BLUF photoreceptors bind an oxidized flavin adenine dinucleotide (FAD) chromophore, which absorbs UV-A and blue light. Photoactivation leads to red-shifted FAD absorption by 10–15 nm,^1,16^ indicating that the FAD remains oxidized, and a hydrogen bond rearrangement around the FAD likely constitutes the photoactivation mechanism.^17−19^ Since the FAD plays crucial roles on the activation/deactivation of the protein, clarification of the static state chemical structures and the reaction dynamics of FAD is essential to understand the photoreaction of BLUF domains.

**Figure 1:**
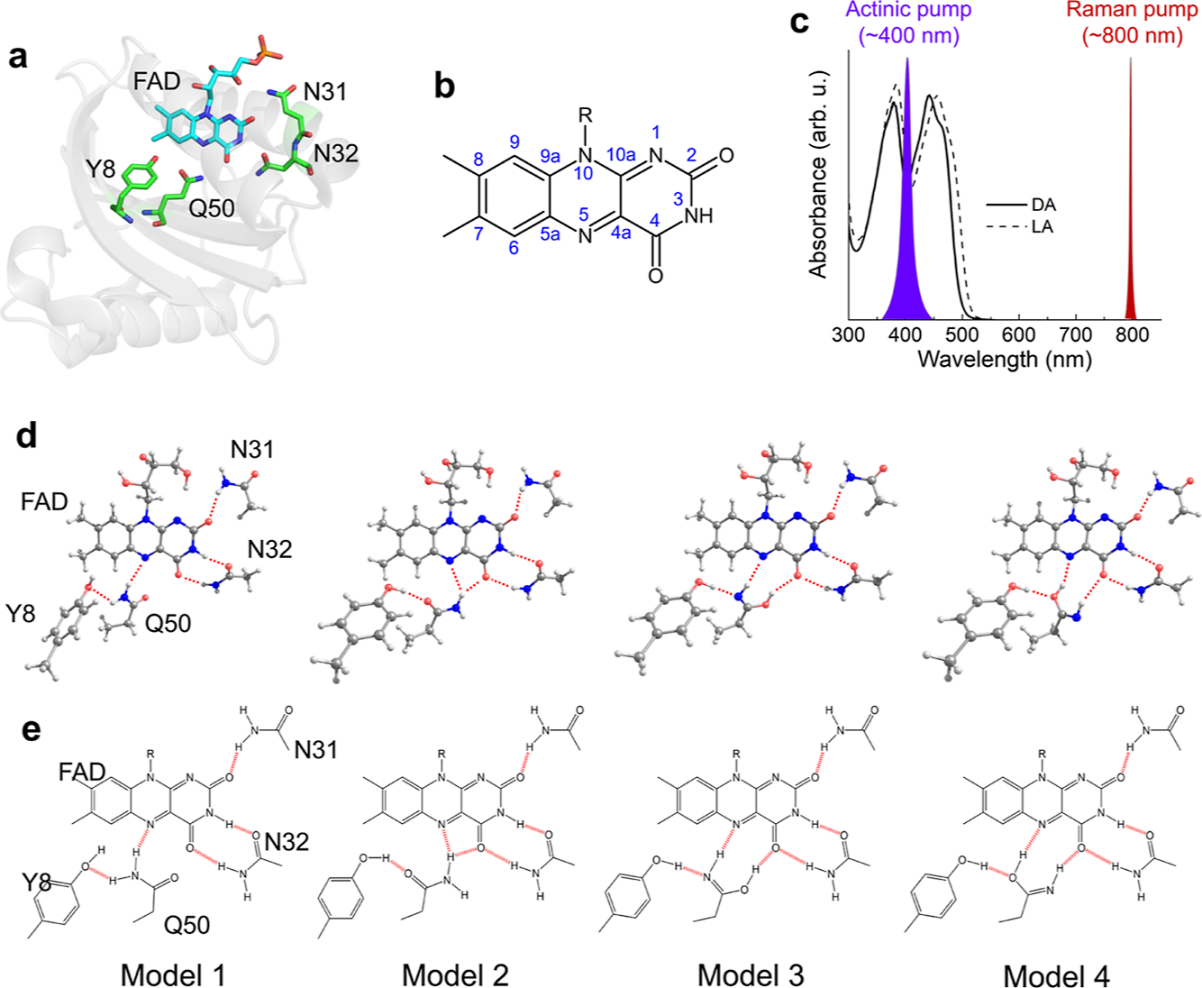
FAD structures and the ground state absorption spectrum of Slr1694. (a) X-ray structure of Slr1694 (PDB: 2HFN).^20^ (b) Chemical structure of FAD with atom numbering. (c) Absorption spectrum of the DA state (solid line) and LA state (dashed line).^21^ A 400 nm actinic pump and an 800 nm Raman pump were selected for the FSRS experiments. (d) Proposed models 1–4 of FAD, Tyr-8, Gln-50, Asn-31, and Asn-32 based on the X-ray structures. Models 3 and 4 correspond to the imidic tautomer of Gln-50. The red dashed lines show hydrogen bonds. (e) Bond structures of models 1–4.

In a representative BLUF photoreceptor, Slr1694 BLUF domain from *Synechocystis* sp. PCC 6803 (also known as SyPixD), Tyr-8 and Gln-50 are involved in the hydrogen bond network near the FAD in the dark-adapted (DA) state (Figure 1).^22^ Upon blue-light illumination to the DA state, the singlet excited (FAD_DA_^*^) state is populated, and sequential electron and proton transfer (*i.e.*, sequential PCET) occurs from Tyr-8 to the FAD. First, FAD^•–^ is formed in a multiphasic fashion in 7, 40, and 180 ps^12,22^ (Figure S1) upon Tyr–FAD electron transfer.^12,23^ Subsequently, proton transfer proceeds in 6 ps, resulting in the formation of FADH^•^.^12,22^ Finally, reoxidation of FAD and a hydrogen bond rearrangement are completed in 65 ps.^12,22^ Similar dynamics involving FAD radicals were observed in the PapB BLUF domain of *Rhodopseudomonas palustris*.^24^ In the *Oscillatoria acuminata* BLUF domain, a concerted PCET reaction on the hundreds of ps to result in FADH^•^ was found.^25^ Remarkably, in the AppA and BlrB BLUF domains from *Rhodobacter sphaeroides*, the hydrogen bond rearrangement proceeds without the apparent involvement of radical species,^26−30^ but it remains unclear whether such species have escaped detection due to kinetic limitations. As a result of the hydrogen-bond switch reaction, conformational changes occur in the BLUF fold on longer timescales^31,32^ that may involve a slide or extension of the β5 strand,^33^ as demonstrated for Slr1694.^34^ This photoactivated or light-activated (LA) state thermally recovers to the DA state in ∼6 s in Slr1694.^35^ When blue-light is applied to the LA state, the singlet excited (FAD_LA_^*^) state is formed, which induces concerted electron and proton transfer from Tyr-8 to FAD in 1 ps, resulting in formation of FADH^•^. The molecules return to the initial LA state in 66 ps^22,36^ (Figure S1).

The hydrogen-bond patterns that connect the FAD to its neighboring residues and their relation to the photodynamics in BLUF photoreceptors have remained controversial, despite many studies with X-ray crystallography,^18,20,37−40^ NMR,^41−44^ Fourier-transform infrared spectroscopy (FTIR),^45−48^ computational studies,^45,49−57^ and time-resolved spectroscopies.^12,23,25,29,30,35,36,58−62^ This issue largely finds its origin in the difficulty in X-ray crystallography to distinguish between O and N nuclei, and the general elusiveness of specific Gln signals in spectroscopy. Figure 1d,e shows four models that may account for hydrogen bond realizations in DA and LA states: model 1 and 2 derive from X-ray crystallographic structures,^18,20,37−39^ while model 3 and 4 were put forward on the basis of computational studies.^49,54^ Note that the positions of a conserved Met and a semiconserved Trp^33,38^ have not been considered here. The hydrogen-bond switch models that have been put forward basically fall into three categories: (i) model 1 and 2 (Figure 1d,e) for DA and LA,^12,18^ briefly referred to here as the “Anderson” hydrogen-bond switch model; (ii) model 2 and 3 for DA and LA,^45^ referred to as the “Domratcheva” hydrogen-bond switch model. Here, model 3 corresponds to the imidic tautomerized form of Gln-50; (iii) model 2 for DA and tautomerized model 4 for LA, referred to as the “Sadeghian/Stelling” hydrogen-bond switch model.^29,54^ In addition, recent free-energy computational studies have suggested that hydrogen-bond realizations of model 1 and 2 might coexist at physiological temperature.^51^ An isotope-labeled FTIR study on the BlrB BLUF domain provided evidence for a tautomerized form of Gln-50 in the LA state^45^ but involved an extensive spectral decomposition procedure and could not discriminate between model 3 and 4. A subsequent study on the AppA BLUF domain found similar evidence for Gln tautomerization in the LA state, with a preference for model 3.^48^ The thermal stability of the high-energy imidic Gln-tautomer was rationalized in a molecular dynamics and quantum mechanical/molecular mechanical (QM/MM) study on the BlrB BLUF domain.^63^

Obviously, the prevailing hydrogen-bond patterns among FAD, Tyr-8, and Gln-50 in DA and LA BLUF domains set the boundaries for the validity of proposed light-induced reaction mechanisms.^12−15,23,29,36,50,54,56,58^ Hence, to deeper understand the static and dynamic structures of the FAD and its adjacent protein side chains, further studies with site-selective sensitivity are required. Femtosecond stimulated Raman spectroscopy (FSRS) is a powerful method to investigate the reaction dynamics of biological chromophores without significant contributions from the apo-protein.^64^ We developed watermarked FSRS to eliminate the notorious baseline problems that have afflicted FSRS since its inception more than a decade ago.^65−71^ Here, the narrowband Raman pump wavelength is varied on a shot-to-shot basis, requiring the fundamental output of the Ti:sapphire amplifier around 800 nm to apply the wavelength modulation and watermarking approach. Because the FAD excited state (FAD*) in BLUF domains exhibits a strong excited-state absorption (ESA) from 600 toward 800 nm,^12,26,36^ its Raman modes are (pre)resonantly enhanced, significantly more so with respect to the FAD ground state and neutral radical modes. Preresonant Raman spectra of the DA and LA states of the BLUF domain electronic ground state were largely identical and gave limited information on the prevailing hydrogen bond patterns in these states.^72^ In contrast, we will show that FAD excited-state Raman spectra obtained by FSRS on the DA and LA state reveal significant differences. The vibrational modes obtained by FSRS can be directly compared with excited-state quantum chemical calculations, giving significant insights into structures of the FAD and its adjacent residues in the static and excited states. Here, we combine watermarked FSRS with QM calculations using the CIS-D3/6-31(d,p) method to investigate the excited-state structures of FAD and its hydrogen bond interactions with Gln-50 and Tyr-8 and potential tautomeric states of Gln. In addition, for a detailed characterization of the key structural changes of the conserved glutamine upon transition to the LA state, we recorded light-minus-dark FTIR difference spectra of heavy isotope-labeled Slr1694 to assess the occurrence of Gln tautomeric states in the photoactivated state.

## Results and Discussion

### FSRS of DA BLUF

**Figure 2:**
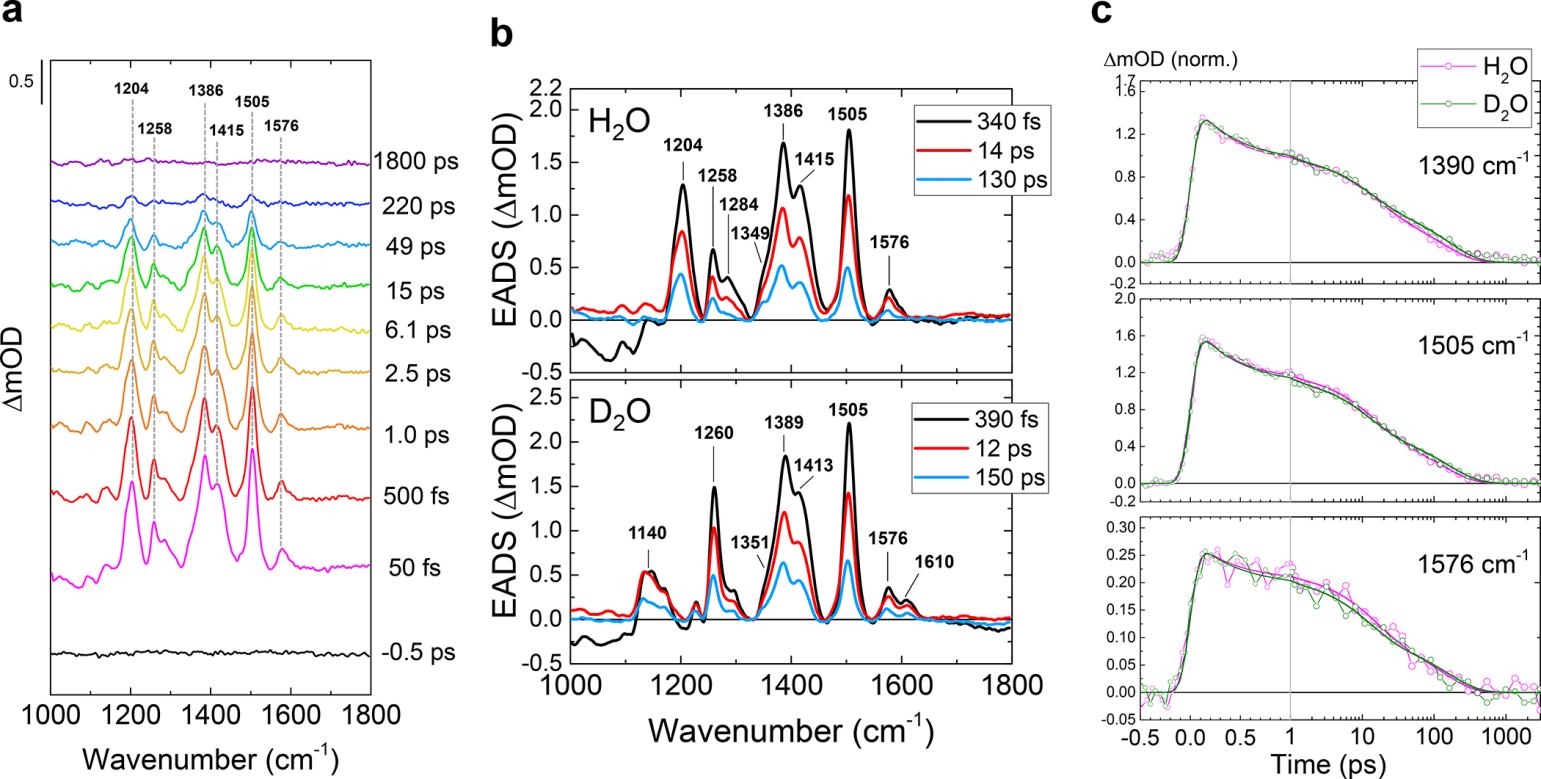
FSRS spectra of DA WT Slr1694. (a) Selected transient Raman spectra. (b) Globally fitted EADS in H_2_O (top) and D_2_O (bottom). (c) Selected time traces with fitting curves. Green and magenta open dots show the raw data in H_2_O and D_2_O, respectively, and the green and magenta lines show the fitting curves in H_2_O and D_2_O, respectively. The time axis is linear up to 1 ps and logarithmic thereafter.

Figure 2a shows selected FSRS spectra of wild-type (WT) Slr1694 BLUF domain in H_2_O buffer of the reaction from the DA state, obtained with a 400 nm actinic pump and an 800 nm Raman pump. The FSRS spectra in H_2_O and D_2_O were globally fitted using a sequential model with increasing time constants, which result in evolution-associated difference spectra (EADS).^73^ We note that sequential analysis and parallel (sum-of-exponents) analysis are mathematically equivalent and yield EADS and decay-associated difference spectra (DADS), with fitted time constants applying to both.^74^ Three time components of 340 fs, 14 ps, and 130 ps were required for a satisfactory fit for Slr1694 in H_2_O, while time constants of 390 fs, 12 ps, and 150 ps were found for Slr1694 in D_2_O (Figure 2b). Figure 2c shows kinetic traces at selected wavenumbers.

Immediately after photoexcitation (Figure 2b, black line), strong peaks appeared at 1204, 1258, 1284, 1349, 1386, 1415, 1505, and 1576 cm^–1^ in H_2_O, similar to FSRS of flavin in solution^69,75^ and the AppA BLUF domain.^76^ These positive peaks decayed with the three time constants, without any significant spectral shifting. The absence of peak shifts indicates that the multi-exponential decays all originate from the same molecular state, that is, the lowest FAD singlet excited state of DA BLUF, denoted FAD_DA_^*^. In transient absorption (TA) and time-resolved fluorescence spectroscopy of the WT Slr1694 BLUF domain, decays of the FAD*_DA_ were observed with three time constants fitted by target analysis: 7, 40, and 180 ps.^12,23^ With the exception of the 310 fs component, the lifetimes of FAD_DA_^*^ observed with FSRS are comparable with the TA results.^12,23^ Strikingly, in contrast to the earlier TA results,^12,35,61^ no signature of the radical intermediates was observed in the FSRS data. Notably, FAD*_DA_ has a strong ESA at wavelengths of >700 nm, whereas only weak or almost no absorption was observed in that region for the FAD^•–^ and FADH^•^ intermediate states.^12^ Probably, the high resonance enhancement with the 800 nm Raman pump of the FAD*_DA_ state overwhelms the signals from the radical intermediate states, and the presence of a long-lived FAD_DA_^*^ additionally precludes their detection. No H/D kinetic isotope effect (KIE) was observed in the decay of FAD_DA_^*^ state (Figure 2b,c), as in TA^12^ and time-resolved fluorescence;^23^ the absence of H/D KIE likely implies that proton transfer is not involved in the decay of the FAD_DA_^*^ state, supporting the sequential electron–proton reaction model that was proposed by Gauden *et al.*(12)

The tri-exponential decay of the FAD_DA_^*^ state with no significant peak shifts indicates that the FAD species excited by the actinic pump have similar structures but varying lifetimes. It was suggested by structure-based free-energy calculations that a heterogeneous hydrogen-bond network near the FAD exists in the ground DA state, which may result in heterogeneous decay of the excited state through distinct electron-transfer pathways.^51^ If this was the case, distinct FAD excited-state Raman features would be expected for the different FAD* decay fractions, resulting from the different hydrogen-bond pattern realizations. However, our FSRS results show that a single FAD species decays in multi-exponential fashion, which indicates that a single realization of the hydrogen-bond pattern between FAD and Tyr-8–Gln-50 underlies the dynamics. The multiple phases in the FAD* decay may be caused by varying donor–acceptor distances in the initial electron transfer. This is supported by the notion that the FAD-binding pocket is highly flexible in BLUF domains.^42^

### FSRS of LA BLUF

To investigate the BLUF photoreaction from the LA state, we used the Slr1694-W91F mutant, which exhibits a ∼50-fold longer-lived LA state than WT (223 *vs* 6 s, for the W91F mutant and WT, respectively)^35^ but fully retains the hydrogen-bond switch reaction of the WT.^35^ The LA state was prepared with backlight blue light-emitting diode (LED) (λ_max_ ∼ 475 nm) illumination. The FSRS spectra of the FAD_DA_^*^ were very similar between WT and the W91F mutant (Figure S2), indicating that the W91F mutation does not affect the Raman properties of the FAD_DA_^*^.

**Figure 3:**
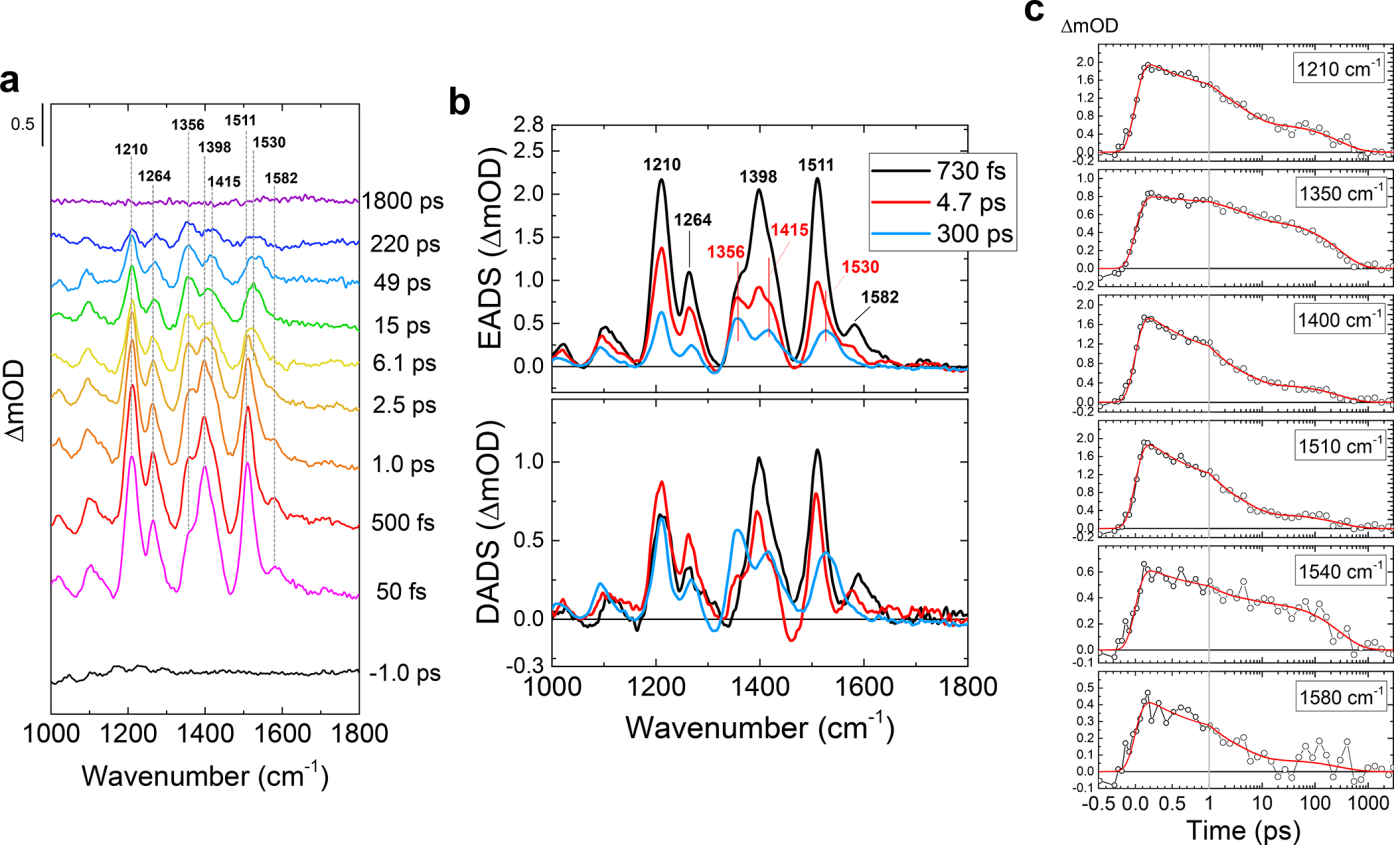
FSRS spectra of the LA Slr1694-W91F mutant in H_2_O. (a) Selected transient Raman spectra. (b) Globally fitted EADS (top) and DADS (bottom). (c) Selected time traces with fitting curves. Open dots show the raw data, and red lines show the fitting curves. The time axis is linear up to 1 ps and logarithmic thereafter.

Figure 3a shows selected transient Raman spectra of the W91F mutant in H_2_O buffer on the reaction from the LA state. The FSRS spectra were globally fitted with three exponential components: 730 fs, 4.7 ps, and 300 ps in H_2_O (Figure 3b) and 470 fs, 3.0 ps and 230 ps in D_2_O (Figure S3). The first EADS (black line, Figure 3b) which can be assigned to the FAD singlet excited state of LA BLUF, denoted FAD_LA_^*^, shows positive peaks at 1210, 1264, 1398, 1511, and 1582 cm^–1^. These peak positions are similar to the FAD_DA_^*^, while slight (∼6 cm^–1^) upshifts were observed for some bands. The second EADS (red line, Figure 3b) shows the Raman spectrum after the first 730 fs decay; the peak intensity at 1398, 1511, and 1582 cm^–1^ significantly dropped, while the 1356 cm^–1^ peak was only slightly weakened. The third EADS (blue line, Figure 3b) shows Raman spectra after the 4.7 ps decay; signals at 1210, 1398, and 1511 cm^–1^ decayed, while peak intensity at 1356 and 1530 cm^–1^ only slightly dropped. The differences of the spectral features are clear in the DADS (lower panel, Figure 3b) and normalized EADS (Figure S4). In 300 ps, all Raman peaks decayed, implying full decay of the molecules that are resonant with the 800 nm Raman pump.

The significant dynamic Raman band shifts of FSRS spectra in the LA state indicate that an intermediate state was formed with a distinct Raman spectrum and was in preresonance with the 800 nm Raman pump. With TA, femto-IR spectroscopy, and target analysis, it was previously shown that FAD_LA_^*^ evolves to FADH^•^ through concerted PCET in 1 ps, with a minor fraction of FAD_LA_^*^ decaying to the initial ground state in 5 ps.^36^ The 730 fs and 4.7 ps components of the FSRS likely correspond to the previously reported 1 and 5 ps decays in TA spectroscopy, respectively. With this assumption, we propose that FADH^•^ Raman spectrum is mixed in the second EADS formed in 730 fs (red line, Figure 3b) and largely constitutes the 300 ps EADS (blue line, Figure 3b). The question arises why such radical FSRS spectral features would appear in the LA state but not in the DA state (Figure 2), given that in the latter case, flavin radicals are clearly involved in the reaction.^12,23,36^ TA data showed that FADH^•^ transiently formed in the DA state does not absorb light at wavelengths >650 nm,^12^ while FADH^•^ from the LA state has a significant absorption tail at >700 nm^36^ and hence has better preresonance with the 800 nm Raman pump. Thus, the long-lived (τ ∼ 300 ps) FSRS species (blue line, Figure 3b) likely corresponds to the stimulated Raman spectrum of FADH^•^ formed from the photoexcited LA state in 730 fs. However, its properties are not entirely clear-cut: its decay time is ∼4.5-fold longer than previously observed in TA (300 *vs* 66 ps), and the Raman spectral signature only partly agrees with that of previously reported FADH^•^ resonant Raman spectra,^21,77^ with the 1530 and 1356 cm^–1^ bands assignable to FADH^•^. The reasons for these discrepancies are not clear but possibly a small fraction of long-lived FAD excited states exists that distorts the spectral signature and lifetime of this component. Note that a long-lived component was required to fit the transient data on the BLUF LA state in our earlier paper.^36^

### Computational Analysis of Excited-State Raman Spectra

The results presented in Figures 2 and 3 demonstrate that the FSRS spectra of the DA and LA state are distinct, with small but significant shifts of the excited-state Raman signatures, while the ground state (pre)resonant Raman spectra of DA and LA BLUF do not show such clear shifts.^72^ We propose that the excited flavin is more sensitive to its interactions with the polar environment because photoexcitation increases the flavin’s dipole moment,^78,79^ and the increased flavin’s polarity enhances interactions with the polar environment. Therefore, the two polar environments would differ more in their interactions with the more polar excited flavin. Having established that the excited-state Raman spectra are sensitive to the precise hydrogen bonding patterns that tether the FAD to the binding pocket in the DA and LA state, the observed differences hence give information about the BLUF reaction model. Though FSRS gives vibrational signals of only the chromophore, FAD, computational analysis allows us to investigate the hydrogen-bond structures of FAD and the nearby amino acids including Glu-50.

To assign the experimentally observed Raman bands to specific hydrogen-bond pattern realizations, we built minimal models of the BLUF active site involving the FAD, Gln-50, Tyr-8, Asn-31, and Asn-32, as shown in Figure 1d,e. Model 1 corresponds to the DA state structure proposed by Anderson *et al.*,^12,18^ with the amino group of Gln-50 oriented toward Tyr-8. Model 2 corresponds to the DA state structure proposed by Kita *et al.* and Jung *et al.*,^37−39^ and the LA state structure proposed by Anderson *et al.*,^12,18^ with the carbonyl of Gln-50 oriented toward Tyr-8. Model 3 represents the imidic tautomerized form of Gln-50 with its N–H group oriented toward Tyr-8 that was proposed as the LA state by Domratcheva and co-workers.^45,49,57^ Finally, model 4 presents the tautomerized form of Gln50 with its O–H group oriented toward Tyr-8 proposed as the LA state by Sadeghian and co-workers^54^ and Stelling and co-workers.^29^

**Figure 4:**
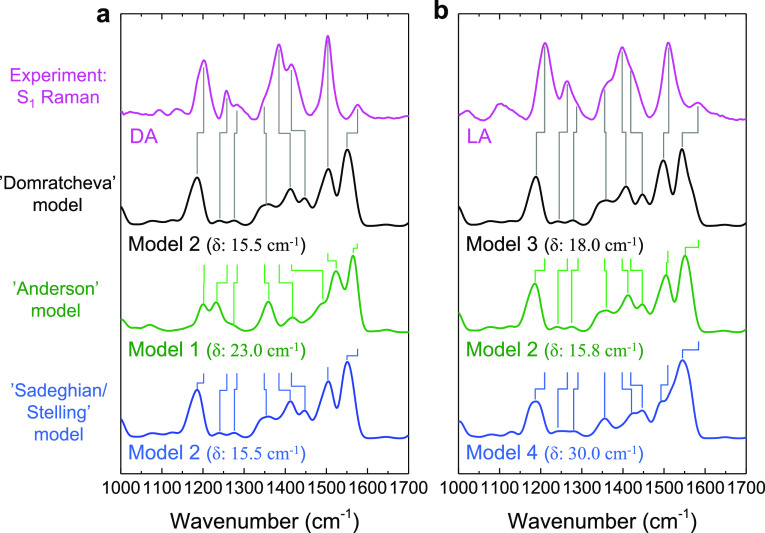
Comparison of calculated and experimental excited S_1_ state Raman spectra in H_2_O for the various hydrogen bond switch models. (a) Second EADS of the DA state (magenta line), reproduced from the upper panel of Figure 2b; calculated Raman signals based on model 2 (black line and blue line) and model 1 (green line). The average deviation of model 1 from the experimental DA state (magenta line) amounts to δ = 23 cm^–1^, whereas that from model 2 amounts to δ = 15.5 cm^–1^. (b) First EADS of the LA state (magenta line), reproduced from the upper panel of Figure 3b; calculated Raman signals based on model 3 (black line), model 2 (green line), and model 4 (blue line). The average deviation of model 3 from the experimental LA state (magenta line) amounts to δ = 18 cm^–1^, whereas that from model 2 amounts to δ = 15.8 cm^–1^ and that from model 2 amounts to δ = 30.0 cm^–1^. The black spectra in (a,b) represent the “Domratcheva” model, the green spectra represent the “Anderson” model, and the blue spectra represent the “Sadeghian/Stelling” model. See Figure 1d,e for the molecular structures of models 1–4. The vertical Raman intensity axis was normalized between the various spectra.

We conducted quantum chemical calculations utilizing the CIS/6-31(dp)-D3 method to determine the Raman-active normal modes in the S_1_ excited state. Previous calculations predicted excited-state vibrational modes of flavin in aqueous solution.^75,80^Tables S1–S4 summarize the calculated frequencies and Raman intensities for models 1–4 in H_2_O and D_2_O, whereas Figures S5–S8 show the atomic motions that constitute the most Raman-active normal modes. Figure 4a,b (black spectra) aims to test the “Domratcheva” hydrogen-bond switch model,^49^ which involves model 2 as the DA state and model 3 as the LA state, to the observed and calculated S_1_ Raman spectra. Figure 4a shows the experimental FAD*_DA_ state (magenta line) and the calculated spectra for model 2 (black and blue lines) and model 1 (green line) convoluted with a spectral width (FWHM) of 30 cm^–1^, with main bands correlated through vertical lines. We estimated the average deviation between experimental and calculated vibrational lines by determining δ = ⟨|ν_exp_ – ν_calc_|⟩ for the eight vibrational bands, that is, the average of the absolute value of the difference between experimental and calculated vibrational frequency. The δ value amounted to 15.5 cm^–1^ for model 2 and 23.0 cm^–1^ for model 1.

We will now discuss the mode assignments indicated by the gray vertical lines considering an earlier FSRS study on isotopically labeled flavin mononucleotide (FMN) in aqueous buffer solution^81^ and experimental and calculated H_2_O/D_2_O exchange effects. In the latter study, S_1_ excited-state modes were identified at 1220, 1389, 1423, and 1507 cm^–1^, similar to the bands at 1204, 1386, 1415, and 1505 cm^–1^ in the current study of DA BLUF, which are correlated with calculated modes at 1190, 1416, 1447, and 1507 cm^–1^, respectively. In ref (81), [U–^15^N_4_], [4,10a-^13^C_2_], [2,4a-^13^C_2_], and [2-^13^C_1_]-FMN were compared with unlabeled FMN (see Figure 1b for flavin atom numbering). In that work, the 1220 cm^–1^ S_1_ band did not exhibit an appreciable shift in the first three labeled samples.^81^ In our work, this band (experimental at 1204 cm^–1^, calculated at 1190 cm^–1^ in model 2, Figure 4a) mainly involves ring I C–H wags (Figure S6), which are indeed not expected to shift upon these isotopic replacements. In contrast, the 1389 cm^–1^ S_1_ band exhibited a significant shift of 14, 13, and 8 cm^–1^ in the first three labeled samples.^81^ In our work, this band (experimental at 1386 cm^–1^, calculated at 1416 cm^–1^ in model 2, Figure 4a) mainly involves the ring II and III C and N atoms in addition to ring I C–H wags (Figure S6) and are thus indeed expected to shift upon these isotopic replacements. The 1423 cm^–1^ S_1_ band was reported to exhibit significant shifts in the first three labeled samples.^81^ In our work, this band (experimental at 1415 cm^–1^, calculated at 1447 cm^–1^ in model 2, Figure 4a) mainly involves ring I C–H wags and methyl rotations (Figure S6), seemingly in contrast with the reported isotopic shifts. However, with inspection of the FSRS data of ref (81), we find that these bands have not been resolved with sufficient accuracy to assign reliable isotopic shifts. We finally consider the 1507 cm^–1^ band in ref (81), which was found to be insensitive to isotope labeling. In our work, this band (experimental at 1505 cm^–1^, calculated at 1507 cm^–1^ in model 2, Figure 4a) mainly involves ring I C–H wags, methyl rotations, and ribityl chain vibrations (Figure S6), which are indeed not expected to shift upon these isotopic replacements.

The experimental band at 1580 cm^–1^ in Figure 4a was not observed in ref (81), precluding any isotopic assessment. Its Raman intensity appears to be significantly overestimated in the calculations. The calculated band is composed of two normal modes at 1561 and 1545 cm^–1^, which correspond to N5–C4a/C10a–N1/N3–H and ring I C8–C9/C9–H normal modes, respectively (Figure S6). In D_2_O, the 1580 cm^–1^ band splits in the experimental spectrum (Figure 2b, lower panel), while in the calculated spectrum, the vibrational frequencies move further apart in D_2_O as well (to 1540 and 1561 cm^–1^, respectively), which supports the assignment of the experimental 1580 cm^–1^ band to these particular modes. Taking together the isotopic and H_2_O/D_2_O exchange effects, we conclude that overall there is reasonably fair agreement between the experimental and calculated spectra, both in frequency (δ = 15.5 cm^–1^) and in Raman intensity.

Figure 4b shows the experimental FAD_LA_^*^ state (magenta line) and the calculated spectra for model 3 (black line), model 2 (green line), and model 4 (blue line), convoluted with a spectral width (FWHM) of 30 cm^–1^, with main bands correlated through vertical lines. As mentioned previously, the overall pattern of the experimental LA state spectrum is similar to that of the DA state, with only minor upshifts of a few bands. Likewise, model 3 shows an overall pattern that is very similar to that of model 2, with only minor shifts. However, model 3 shows small downshifts with respect to model 2, whereas the experimental LA state shows slight upshifts with respect to the DA state. Here, δ = 18 cm^–1^ for model 3, 15.8 cm^–1^ for model 2, and 30.0 cm^–1^ for model 4.

In a similar fashion, the green spectra in Figure 4a,b examine the “Anderson” hydrogen-bond switch model, which involves model 1 as DA and model 2 as LA.^12,18^Figure 4a shows the calculated spectrum for model 1 (green line) and the experimental FAD_DA_^*^ state (magenta line). Notably, the overall calculated Raman intensity for model 1 is lower than that for model 2 and 3 (Tables S1–S3) and shows an overall different pattern of Raman band intensities and frequencies. Thus, this hydrogen-bond switch model (model 1 for DA, model 2 for LA) predicts a relatively large change in the vibrational band pattern between DA and LA states, which is not experimentally observed. As a consequence, the agreement between model 1 and the experimental DA Raman intensities and frequencies is rather poor, which is also indicated by a larger δ = 23 cm^–1^ in model 1 as compared to that of model 2, where δ = 15.5 cm^–1^. The experimental BLUF FAD_LA_^*^ state (Figure 4b, magenta line) agrees quite well with model 2 (Figure 4b, green line), with δ = 15.8 cm^–1^.

The blue spectra in Figure 4a,b examine the “Sadeghian/Stelling” hydrogen-bond switch model, comparing calculated model 4 with the experimental LA spectrum (note that the black and blue spectra are identical in Figure 4a). The overall calculated band pattern of model 4 is quite distinct from that of model 3 and resembles the experimental spectrum significantly less as compared to model 3, with δ = 30 cm^–1^ for model 4 as compared to δ = 18 cm^–1^ for model 3. In addition, as for the “Anderson” model, the “Sadeghian/Stelling” hydrogen-bond switch model (model 2 for DA, model 4 for LA) predicts a relatively large change in the vibrational band pattern between DA and LA states, which is not experimentally observed.

Based on the considerations above, we may relate the agreement, or lack thereof, between experimental and calculated S_1_ state Raman spectra to the hydrogen-bond patterns and tautomeric states in the BLUF active site of model 1–4. The initially proposed “Anderson” hydrogen-switch model, which features model 1 for the DA and model 2 for the LA state shows large discrepancies: the calculated spectra would predict large differences between the DA and LA states (Figure 4a,b, green lines), which are not experimentally observed. In addition, the agreement between model 1 and the experimental DA spectrum is rather poor (δ = 23 cm^–1^). For the “Sadeghian/Stelling” hydrogen-bond switch model, similar problems apply: here too, the calculated spectra would predict large differences between the DA and LA states (Figure 4a,b, blue lines), which are not experimentally observed. Also, there is poor agreement between model 4 and the experimental LA spectrum (δ = 30 cm^–1^).

On the other hand, the “Domratcheva” hydrogen-bond switch model, which features model 2 for the DA state and model 3 for the light state, shows overall fair agreement, in calculated Raman intensity and frequencies (Figure 4a,b, black lines, δ = 15.5 cm^–1^ for DA and 18 cm^–1^ for LA). Although model 3 predicts overall slightly downshifted bands with respect to model 2, contrary to experimental observation, in absolute terms the “Domratcheva” model is clearly superior to the “Anderson” and “Stelling/Sadeghian” hydrogen-bond switch models. Hence, we conclude, within the accuracy of our computational approach, that FSRS spectroscopy of the Slr1694 BLUF domain reveals that the “Domratcheva” model likely represents the most correct model for BLUF photoactivation.

H_2_O/D_2_O effects on the excited-state Raman spectra in DA and LA BLUF domains are described in the Supporting Information.

### Characterization of the Light-Induced Structural Changes *via* FTIR Spectroscopy and Selective Isotope-Labeling

For a detailed characterization of the key structural changes of the conserved glutamine upon transition to the LA state, we recorded light-minus-dark FTIR difference spectra of heavy isotope-labeled Slr1694. Site-specific isotope labeling supports the interpretation of these highly complex spectra by introducing site- or group-specific shifts in the molecular vibration frequency of the corresponding bonds. A previous FTIR study on BlrB labeled with ^15^N at the ε-position of glutamine residues combined with quantum chemical calculations illustrated a contribution of both the flavin and glutamine to the major light state signal, composed of the flavin C4=O stretch vibration peaking at 1697 cm^–1^ and a glutamine-associated C=N stretch vibration signal at around 1691 cm^–1^, thus providing support for a glutamine tautomer being formed in the light state of BlrB.^45^ Iwata and co-workers provided further proof for a glutamine tautomer being formed upon photoactivation of BLUF domains by a similar isotope labeling study on the BLUF domain of the AppA photoreceptor.^48^ They combined ^15^N glutamine labeling of the AppA BLUF domain with a reconstitution of the protein with uniform ^13^C-labeled flavin chromophore to remove the dominant absorption of the isoalloxazine carbonyl vibrations.

**Figure 5:**
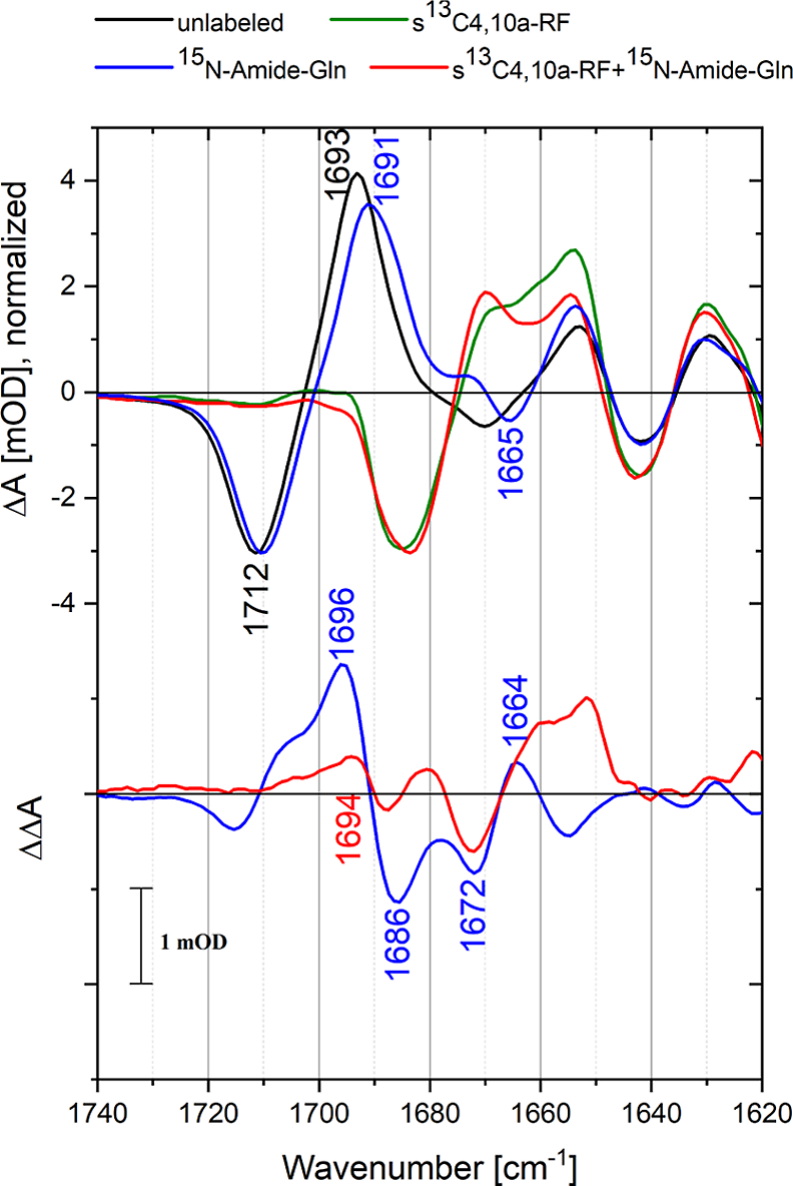
Light-minus-dark FTIR difference spectra of unlabeled (black), ^15^N-amide glutamine-labeled (blue), ^13^C4/C10a flavin-labeled (green), and ^15^N-amide glutamine/^13^C4/C10a flavin-labeled Slr1694 (red) in the amide I spectral region. The lower panel shows the double differences of unlabeled-minus-labeled spectra of completely unlabeled minus ^15^N-amide glutamine-labeled difference spectra (blue) and flavin-labeled minus flavin and glutamine-labeled difference spectra (red). Both double-difference spectra show a signal at ∼1695 cm^–1^ that loses intensity upon glutamine labeling.

Here, we pursued a similar strategy for Slr1694 (Figure 5). Compared to the aforementioned double-labeling approach for the AppA BLUF domain, we introduced site-specific isotope ^13^C labels in the isoalloxazine core of the chromophore at the C4 and C10a position. The labeling of the chromophore and the glutamine was accomplished without *in vitro* reconstitution of the protein through the production of the protein in a flavin and glutamine double auxotroph *Escherichia coli* strain.

The light-minus-dark FTIR spectrum (Δ*A* spectrum) of unlabeled Slr1694 (Figure 5, black line) is dominated by a strong feature at 1710(−)/1693(+) cm^–1^, which is primarily assigned to a downshift of the FAD C4=O mode.^17^ The 1667(−)/1652(+) cm^–1^ feature is assigned to an upshift of the FAD C2=O mode,^17^ possibly in combination with a bleach of the Gln-50 C=O mode.^23^ The positive feature at 1630 cm^–1^ has been assigned to an extension of the β5 strand of the BLUF domain β-sheet with Trp-91 upon photoactivation.^34^ The ^15^N-amide glutamine labeling induces a small downshift of the large positive band at 1693 cm^–1^ and of the negative band at 1712 cm^–1^ The double-difference spectrum (ΔΔ*A*, Figure 5, bottom panel, blue line) is defined as the unlabeled Δ*A* spectrum minus the labeled Δ*A* spectrum.

The ΔΔ*A* spectrum in ^15^N-Gln-labeled BLUF domain is dominated by a positive band at 1696 cm^–1^ accompanied by a negative band at 1686 cm^–1^ and further shows a negative/positive band pair at 1672/1664 cm^–1^ and a small negative band at 1712 cm^–1^ with a positive shoulder at 1705 cm^–1^. Notably, the band pattern in the ΔΔ*A* spectrum is similar to that observed before in the BlrB BLUF domain.^45^ The dominant 1696(+)/1686(−) cm^–1^ band pair corresponds to that of BlrB at 1697(+)/1689(−) cm^–1^, where it was mainly assigned to a flavin C4=O frequency downshift in the light state upon ^15^N Gln labeling.^45^ The 1672 (−)/1664(+) cm^–1^ band pair corresponds to that at 1667(−)/1660(+) cm^–1^ of BlrB, where it was assigned to the Gln C=O vibration in the dark state downshifting upon ^15^N Gln labeling, with the C=N band of ^15^N-labeled Gln tautomer (−) in the light state contributing to the negative band. The 1712(−)/1705 (+, shoulder) cm^–1^ band pair is clearly related to a downshift of the flavin C4=O vibration in the dark state upon ^15^N Gln labeling.

As noted in ref (45), the putative C=N signature of the Gln tautomeric state was largely obscured by ^15^N Gln labeling effects on the flavin C4=O vibration, and the former signal could only be extracted through spectral modeling of partly compensating positive and negative features, rendering this procedure rather sensitive to baseline and normalization uncertainties in the constructed ΔΔ*A* spectra. To eliminate the pronounced effects of the FAD C4=O band on the FTIR (double-) difference spectra, we took a double-labeling approach by creating single-labeled Slr1694 BLUF domains with ^13^C4/C10a flavin and double-labeled Slr1694 BLUF domains with ^13^C4/C10a flavin and ^15^N-amide glutamine. In these samples, the flavin C4=O signals are expected to shift outside the spectral window where the Gln tautomeric C=N stretch is anticipated to absorb. In the single-labeled FTIR difference spectrum (Figure 5, top panel, green line), the large flavin C4=O negative/positive feature indeed downshifted by ∼32 to 1680(−)/1663(+) cm^–1^, leaving only very small amplitude signals in the region 1690–1720 cm^–1^. In the double-labeled FTIR difference spectrum (Figure 5, top panel, red line), a small difference with the single-labeled sample is again observed in the region around 1690 and around 1670 cm^–1^. The corresponding double-difference spectrum (ΔΔ*A*, bottom panel, Figure 5, red line) shows a positive band at 1694 cm^–1^, accompanied by a negative band at 1672 cm^–1^ and a positive band near 1660 cm^–1^. In addition, a minor 1686(−)/1680(+) cm^–1^ feature is observed in the ΔΔ*A* spectrum. Strikingly, the large 1696(+)/1686(−) cm^–1^ band pair signal of the ^15^N glutamine labeling experiment with unlabeled flavin (Figure 5, bottom panel, black line) assigned to flavin C4=O in the light state^45^ has mostly disappeared in the double-labeling experiment, and only a smaller positive signal at 1694 cm^–1^ is left. The latter signal may now be unambiguously assigned to the Gln C=N tautomeric stretch vibration, and hence, this experiment demonstrates that in the ^15^N Gln labeling experiment with unlabeled flavin, the Gln tautomeric C=N stretch vibration signal indeed is overwhelmed by the 1696(+)/1686(−) cm^–1^ flavin C4=O signal, consistent with the analysis of ref (45). The negative band at 1672 cm^–1^ may be assigned to a superposition of the Gln C=O vibration in the dark state and the ^15^N-labeled Gln tautomer C=N band in the light state, which has downshifted by 22 cm^–1^ upon ^15^N labeling. The positive 1664 cm^–1^ band is then due to the C=O band of ^15^N-labeled Gln in the dark state, representing a modest isotopic shift of ∼8 cm^–1^. Indeed, the 1672(−)/1664(+) feature is preserved between the single-labeled and double-labeled ΔΔ*A* spectra (blue and red lines in Figure 5, bottom panel, respectively), consistent with this interpretation and in accordance with ref (45) Finally, the minor 1686 (−)/1680(+) cm^–1^ feature in the ΔΔ*A* spectrum with double labeling (red line in Figure 5, bottom panel) is the counterpart of the 1712/1705 cm^–1^ feature of the ΔΔ*A* spectrum with single labeling (blue line in Figure 5, bottom panel) and is due to a downshift of the labeled flavin C4=O band in the dark state upon ^15^N Gln labeling, as becomes apparent from inspection of the corresponding Δ*A* spectra.

In summary, in agreement with the work by Domratcheva *et al.*(45) and Iwata *et al.*,^48^ we assign the ∼1694 cm^–1^ band in the double difference spectra to the Gln-50 C=N stretching mode in the light state of Slr1694. The negative band at 1672 cm^–1^ observed in both ΔΔ*A* spectra belongs to this mode in the ^15^N Gln-labeled samples. Concomitantly, we observe the disappearance of the Gln-50 C=O mode at 1672 cm^–1^ upon photoactivation, consistent with our earlier tentative assignment,^23^ and its downshift to 1664 cm^–1^ upon ^15^N isotopic labeling. Hence, this work provides strong evidence for Gln-50 tautomerization in the photoactivated state in the Slr1694 BLUF domain.

In the results on the AppA BLUF domain by Iwata *et al.*,^48^ a feature at 1698(+)/1689(−) cm^–1^ in their ΔΔA spectrum of ^15^N Gln-labeled samples was observed, similar to the present study and ref (45). However, the negative/positive features near 1672/1664 and 1712/1705 cm^–1^ of the present study and that of ref (45) were not detected. In addition, contrary to the present study, in a double-labeling approach with ^13^C-labeled flavin and ^15^N-labeled Gln, a tautomeric Gln C=N band was found at 1683 cm^–1^ rather than that at 1698–1694 cm^–1^. These discrepancies may be partly due the different scaling procedures of the constituent Δ*A* spectra. In addition, these discrepancies might hint at differences in the molecular nature of different BLUF domains or occur due to differences in sample preparation techniques: BlrB and Slr1694 were labeled during protein production, whereas the AppA protein was reconstituted with ^13^C-labeled FMN after purification. Differences in the labeling degree of the glutamine—the labeling efficiency for Slr1694 is lower than what was reported for AppA—might lead to weaker signals but are unlikely to account for such stark differences.

### Merits of the Various Hydrogen-Bond Switch Models

In this study, we have assessed the hydrogen bond patterns that connect FAD, Gln-50, Tyr-8, and the Gln-50 orientation and tautomeric states in the DA and LA states of the Slr1694 BLUF domain by a combination of FSRS, quantum-chemical methods, and FTIR on specifically isotope-labeled samples. We find that the FSRS spectra of the DA and LA states are mostly consistent with the “Domratcheva” hydrogen bond switch model, where the oxygen of Gln-50 hydrogen bonds with Tyr-8 in the DA state, and Gln rotates and tautomerizes to the imidic form in the LA state. In support of this model, we directly identified the C=N stretch of the Gln tautomer in the LA state using FTIR double-difference spectroscopy with specific isotope labeling of Gln and FAD. Hence, we consider this hydrogen bond switch model as the most likely, consistent with other FTIR work^45,48^ and recent QM/MM molecular dynamics studies.^15^ Still, relating the BLUF DA state to model 2 in Figure 1d,e poses a conundrum: it was shown by NMR spectroscopy that the Tyr-OH proton rapidly exchanges in the DA state,^41^ which is consistent with the dynamic flexible nature of FAD-Gln-Tyr,^42−44^ but difficult to understand in terms of the strong hydrogen bond between Tyr-OH and Gln C=O in the Kita and Jung X-ray structures.^37−39^ Possibly, the flexibility of FAD-Gln-Tyr is significantly reduced in the crystalline state.

The early hydrogen-bond switch model by Anderson *et al.*(18) was initially supported by the observation of strong localization of the Tyr-OH proton in the LA state,^41^ resulting in an unusually strong hydrogen bonding^47^ which seemed most consistent with a Tyr-OH···O=C structure involving two strongly electronegative oxygen nuclei in the LA state.^36^ However, strong hydrogen bonding between Tyr-OH and the N−H of the imidic tautomer of Gln-50 was qualitatively reproduced in ref (57), weakening such line of argumentation. A first kinetic model of the Slr1694 photoreaction published by some of us (J.T.M.K., T.M., and P.H.), based on the “Anderson” model and involving sequential electron and proton transfer followed by radical-pair recombination as observed with ultrafast spectroscopy initially gained traction,^12,23,61^ but it was later found that alternative reaction models involving Gln tautomeric states could also account for the ultrafast spectroscopic observations.^13−15^ Hence, with the present and earlier^48,49^ results, the“Anderson” hydrogen bond switch model can effectively be ruled out.

In the “Sadeghian/Stelling” hydrogen-bond switch model,^29,54^ Gln-50 is proposed to form a tautomer in the LA state but without rotating its side chain. It is very difficult to spectroscopically distinguish the orientation of this imidic tautomer (Figure 1d,e, model 4) from that of the rotated tautomer (Figure 1d,e, model 3) using FTIR spectroscopy: our FTIR data nor that of ref (45) provide sufficient information to that end. However, our FSRS data of the LA state are less compatible with the calculated S_1_ Raman spectrum of model 4, which leads us to conclude that this hydrogen-bond switch model is less likely to apply, consistent with the findings of Iwata *et al.*(48)

## Conclusions

In this study, excited-state chemical structures of/near the FAD and its excited-state dynamics of the DA and LA states were investigated by FSRS combined with computational calculation based on the CIS-D3/6-31(d,p) method. In the DA state, the excited FAD_DA_^*^ state decayed in 340 fs, 14 ps, and 130 ps. Since the resonance with the 800 nm Raman pump was weak in other states than the FAD_DA_^*^ state, FSRS only showed pure FAD_DA_^*^ state signals. No significant band shifts were observed during the FAD_DA_^*^ decays, implying that the FAD_DA_^*^ state is homogeneous with regard to the hydrogen-bond pattern. On the reaction from the LA state, which was studied with the W91F mutant, overall similar excited FAD_LA_^*^ state Raman features as compared to FAD_DA_^*^ were observed, with slight but significant differences from the FAD_DA_^*^ state. We assigned the FSRS spectra of the FAD excited state in DA and LA utilizing quantum chemical calculations. The experimental excited-state Raman spectra of DA and LA BLUF can be assigned correspondingly to model 2 and model 3 (Figure 1d,e), respectively, but not to model 1 or 4. This observation implies that the “Domratcheva” hydrogen-bond switch model, which involves tautomerization of Gln-50 to the imidic form accompanied by side-chain rotation in the LA state,^49^ is more likely to apply to the Slr1694 BLUF photoreceptor. This finding is corroborated by FTIR experiments with specific double-isotope labeling of Gln and FAD, where in the difference spectra and double-difference spectra, the C=N stretch band of the imidic Gln-50 tautomer was identified in the LA state and disentangled from the vibrationally coupled FAD C4=O mode.

## Methods

### Sample Preparation for FSRS

WT Slr1694 BLUF photoreceptors and the W91F mutant samples were prepared, as reported previously.^12,35,36^ The samples were filled in a homemade sample holder that has two 2 mm thick CaF_2_ plates. The sample thickness was adjusted to 200 μm with an appropriate sample spacer. The sample holder was set on a Lissajous scanner that ensures rapid sample refreshment with a time interval of 60 s between successive exposures to the laser pulses.^82^

### Femtosecond-Stimulated Raman Spectroscopy

Femtosecond time-resolved-stimulated Raman experiments were performed with the watermarked stimulated Raman setup reported previously.^65−68^ Raman pump (∼800 nm, ∼10 μJ) and Raman probe (∼840–960 nm) were spatiotemporally overlapped at the sample position with a diameter of ∼100 μm. The actinic pump (∼400 nm, ∼2 μJ) was focused on the protein sample to a diameter of ∼150 μm with a time delay from −50 to 1800 ps at 63 data points (logarithmically spaced after 2 ps), generated by an optical delay line. The Raman pump passed through a specially designed chopper blade for the watermarking approach,^65^ which produces 14 Raman pump sequences whose wavelengths slightly shifted each other. As a result, 14 different stimulated Raman experiments are effectively performed simultaneously, which enables the nearly baseline-free watermarking approach. The sample exposure time to the beams was ∼1 h in total for each time-resolved stimulated Raman experiment. For experiments of the LA state of the Slr1694-W91F mutant, 475 nm LED (∼10 mW/cm^2^) was continuously irradiated to the sample during the experiments. Residual baselines of FSRS were manually corrected before global analysis.

### Global Analysis Methodology

Global analysis was performed for the FSRS spectra using the Glotaran program.^83,84^ With global analysis, all wavenumbers were analyzed simultaneously with a set of common time constants.^73^ A kinetic model was applied consisting of sequentially interconverting, EADS, that is, 1 → 2 → ... in which the arrows indicate successive mono-exponential decays of a time constant, which can be regarded as the lifetime of each EADS.^73^ The first EADS corresponds to the difference spectrum at time zero. The first EADS evolves into the second EADS with time constant τ_1_, which in turn evolves into the third EADS with time constant τ_2_, and so on. The procedure clearly visualizes the evolution of the intermediate states of the protein.^85^ We note that sequential analysis and parallel (sum-of-exponents) analysis are mathematically equivalent and yield EADS and DADS, with fitted time constants applying to both.^86^ The standard errors in the time constants were less than 10%.

### Computational Methods

Molecular complexes mimicking hydrogen-bonding interactions of the flavin cofactor with residues of the active site (Tyr-8, Gln-50, Asn-34, and Asn-35) were computationally characterized to facilitate the assignment of the Raman spectra. Starting atomic coordinates of the models were assigned according to the PDB model 2HFN (molecule A). Geometry optimization, vibrational analysis in harmonic approximation, and calculations of Raman intensities were performed using the CIS-D3/6-31(d,p) method in the S_1_ state dominated by the highest occupied molecular orbital–lowest un-occupied molecular orbital ππ* excitation of the isoalloxazine chromophore; the harmonic vibrational frequencies were scaled by 0.91. In order to model the D_2_O effect, the computations of the Raman spectra were re-computed for models in which the hydrogen atoms of the OH and NH/NH_2_ groups were substituted by deuterium atoms. The calculations were performed using the Firefly quantum-chemistry program, version 8.2^87^ which is partially based on the US GAMESS source code.^88^

### Construction of the *E. coli* Strain Glutamator and pET21a-slr1694

The newly developed expression strain derives from the glutamine and riboflavin auxotrophic parent CpXFΔQv2^89^ and shows a strikingly reduced glutamine requirement of 50% compared to its ancestor. This characteristic results from additional deletions of the genes *ybaS*, *gltB*, and *yneH.* They were gradually removed by using the one-step inactivation method^90,91^ to reduce the isotope scrambling of glutamine further. Table S5 shows the primer pairs utilized for the knock-out and verification of the correct deletion scar. The resulting strain harbors a chromosomal Kanamycin resistance as it was impossible to eliminate the encoding cassette after the last knock-out of *yneH*. Therefore, the *slr1694* open reading frame was transferred from pET28a-slr1694 into the ampicillin selectable vector pET21a(+) *via* digestion with *Xba*I and *Xho*I and subsequent ligation.

### ^15^N-amide-glutamine Labeling of Slr1694

A global labeling pattern was achieved by using the auxotrophic *E. coli* strain CpXΔQv2.^89^ Cells were transformed with pET28a-slr1694, and positive clones were cultivated under high cell density conditions^3^ in 500 mL of minimal medium supplemented with 1% ^15^N-amide-l-glutamine (98% atom., CortecNet). 24 mg/mL protein was purified by immobilized metal ion affinity chromatography. The labeling degree was determined by mass spectrometry and estimated to reach predominantly ∼10%. It varied among the detected peptides, which covered 26% of the whole protein.

### ^15^N-amide-glutamine and ^13^C4,10a-Riboflavin Double Labeling of Slr1694

After transformation of the glutamator with pET21a-slr1694, protein expression was carried out as mentioned above with supplementation of 20 μM ^13^C4,10a-riboflavin, 0.05%(w/w) ^15^N-amide-l-glutamine (98% atom., CortecNet) per gram glucose, and 0.02% (w/v) of the remaining amino acids (except l-tyrosine and l-cysteine). Purification yielded 28 mg/mL protein, and mass spectrometry revealed a labeling degree of predominantly ca. 10%, differing between peptides, which covered 42% of the whole protein.

### FTIR Spectroscopy

FTIR spectroscopy was performed, as described.^89^ Protein samples were concentrated to an OD_441nm_ ∼ 70–100, and ∼2–5 μL was placed between two CaF_2_ plates without spacer and sealed with silicon grease for tightness. FTIR spectra between 1800 and 1000 cm^–1^ were recorded using a Bruker IFS66s spectrometer with 3 cm^–1^ resolution. Light-minus-dark difference spectra were generated by recording 100 scans of background without application of blue light and 100 scans with application of blue light (LED Luxeon, 1 W, 460 nm). To estimate the experimental drifting during the measurement, 100 scans of background and 100 scans of sample were recorded without application of blue light to generate a dark-minus-dark difference spectrum. 10 experiments of each light-minus-dark and dark-minus-dark spectra were averaged, and the resulting data sets were substracted to correct for the experimental drift.

## Supporting Information Available

H_2_O/D_2_O effects, previously reported reaction models, additional FSRS spectra comparison, illustrations for vibrational modes, and frequencies and Raman activities for computational models (PDF)
