# Supplementary material for: Spectroscopic and Computational Observation of Glutamine Tautomerization in the Blue Light Sensing Using Flavin Domain Photoreaction
Source: J Am Chem Soc. 2023 Jan 6;145(2):1040–52. doi: 10.1021/jacs.2c10621 (PMC9853863; doi:10.1021/jacs.2c10621)
Supplement: Supplementary file 1 — ja2c10621_si_001.pdf [file ja2c10621_si_001.pdf]

Supporting information to

Spectroscopic and Computational Observation of Glutamine Tautomerization in the Blue Light sensing Using Flavin Domain Photoreaction

Yusaku Hontani[1,¶,†], Jennifer Mehlhorn[2,¶], Tatiana Domratcheva[3,4], Sebastian Beck[5], Miroslav Kloz[1,6], Peter Hegemann[2], Tilo Mathes[1,2] and John T.M. Kennis[1*]

[1]Department of Physics and Astronomy, Vrije Universiteit Amsterdam, Amsterdam 1081 HV, De Boelelaan, The Netherlands

[2]Institut für Biologie, Experimentelle Biophysik, Humboldt Universität zu Berlin, Invalidenstrasse 42, D-10115 Berlin, Germany

[3]Department of Biomolecular Mechanisms, Max Planck Institute for Medical Research, 69120 Heidelberg, Germany

[4]Lomonosov Moscow State University, Department of Chemistry, Moscow 119991, Russia

[5]Department of Chemistry, Humboldt-Universität zu Berlin, Brook-Taylor-Str. 2, 12489 Berlin, Germany

[6]ELI-Beamlines, Institute of Physics, Na Slovance 2, 182 21 Praha 8, Czech Republic

[¶] these authors contributed equally

*Corresponding author: j.t.m.kennis@vu.nl

[†]Current address: Brain Research Institute, University of Zurich, 8057 Zurich, Switzerland

Supplemental Note

H_2O/D_2O effects in the FSRS spectra of DA and LA BLUF domains

The bottom panel of **Fig. 2b** shows the EADS of the FSRS spectra of the DA BLUF domain in D_2O. The overall pattern of vibrational bands is similar to that of the DA BLUF domain in H_2O, with main bands at 1140, 1260, 1351, 1389, 1413, 1505, 1576 and 1610 cm^{-1}. With respect to BLUF in H_2O, the large band at 1204 cm^{-1} has largely disappeared and a new band at 1140 cm^{-1} has come up, while the band at 1258 cm^{-1} in H_2O has gained intensity in D_2O, now peaking at 1260 cm^{-1}. In addition, the band at 1576 cm^{-1} has split in two, with bands at 1576 and 1610 cm^{-1}. The remainder of the vibrational pattern is nearly the same as in H_2O, involving only small shifts. A very similar pattern of band alterations was previously observed for FAD in aqueous solution, in H_2O and D_2O.[1]

According to the calculations for Model 2, the experimentally observed large band at 1204 cm^{-1} observed in H_2O that downshifts to 1140 cm^{-1} and loses intensity in D_2O, is a composite of modes at 1170 and 1190 cm^{-1}, and mainly correspond to C_9-H and C_6-H in-plane bending modes, respectively (**Fig. S6**). Indeed, the calculated 1170 cm^{-1} mode shifts down to 1133 – 1144 cm^{-1} and loses intensity in D_2O (Table S2), as observed in the experimental spectra. However, the calculated 1190 cm^{-1} mode shifts up slightly to 1192 cm^{-1} and maintains intensity in D_2O, which does not agree with the experimental results. As mentioned earlier in this manuscript, the splitting of the experimental 1576 cm^{-1} band into 1576 and 1610 cm^{-1} is supported by the calculations, which indicate that it is composed of bands at 1545 and 1561 cm^{-1}, which move apart in D_2O (Table S2).

The other major difference between DA BLUF in H_2O versus D_2O is the intensity gain of the 1260 cm^{-1} band in D_2O as compared to H_2O. In H_2O, this mode likely corresponds to the calculated modes between 1236 - 1276 cm^{-1}, which have rather low intensity. In the calculated D_2O spectra, no obvious intensity gain is predicted in this spectral region (Table S2). We note that essentially the same intensity gain effect for a band at 1258 cm^{-1} was observed for FAD in aqueous solution.[1] In that work, this intensity gain was assigned to mixing in of a N_3-D wagging vibration at this wavenumber, on the basis of TD-B3LYP/TZVP normal-mode analysis.

Fig. S3 shows the FSRS spectra for LA BLUF domains in H_2O (upper panel) and D_2O (lower panel). The differences between H_2O and D_2O are very similar to those observed for DA BLUF.

Likewise, the differences in calculated frequencies and intensities are very similar between model 2 and 3 in H_2O and D_2O, implying that the above considerations for DA BLUF apply to LA BLUF as well.

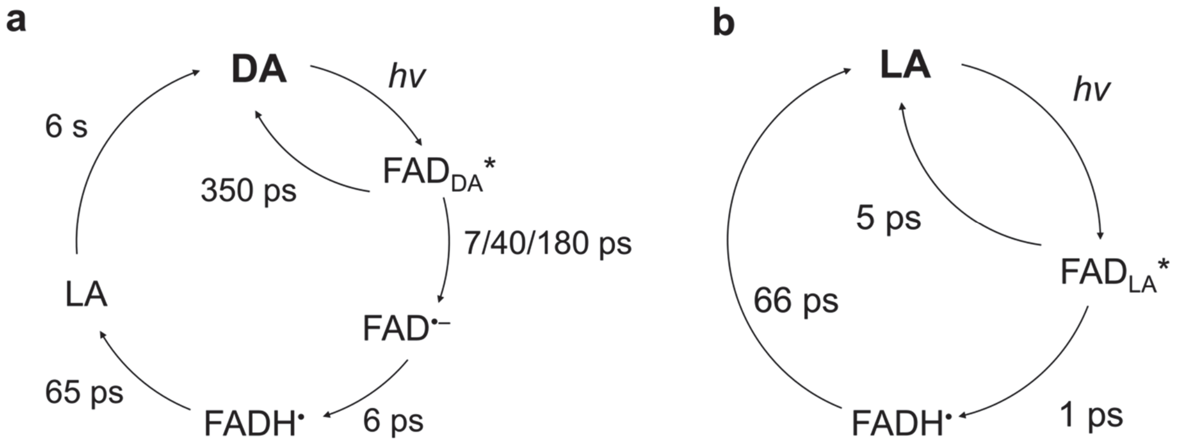

Figure S1. Previously proposed reaction models of Slr1694 BLUF photoreceptor. Photoreaction models of (**a**) dark-adapted (DA) state proposed by Gauden *et al.*[2] and (**b**) light-adapted (LA) state proposed by Mathes *et al*.[3]

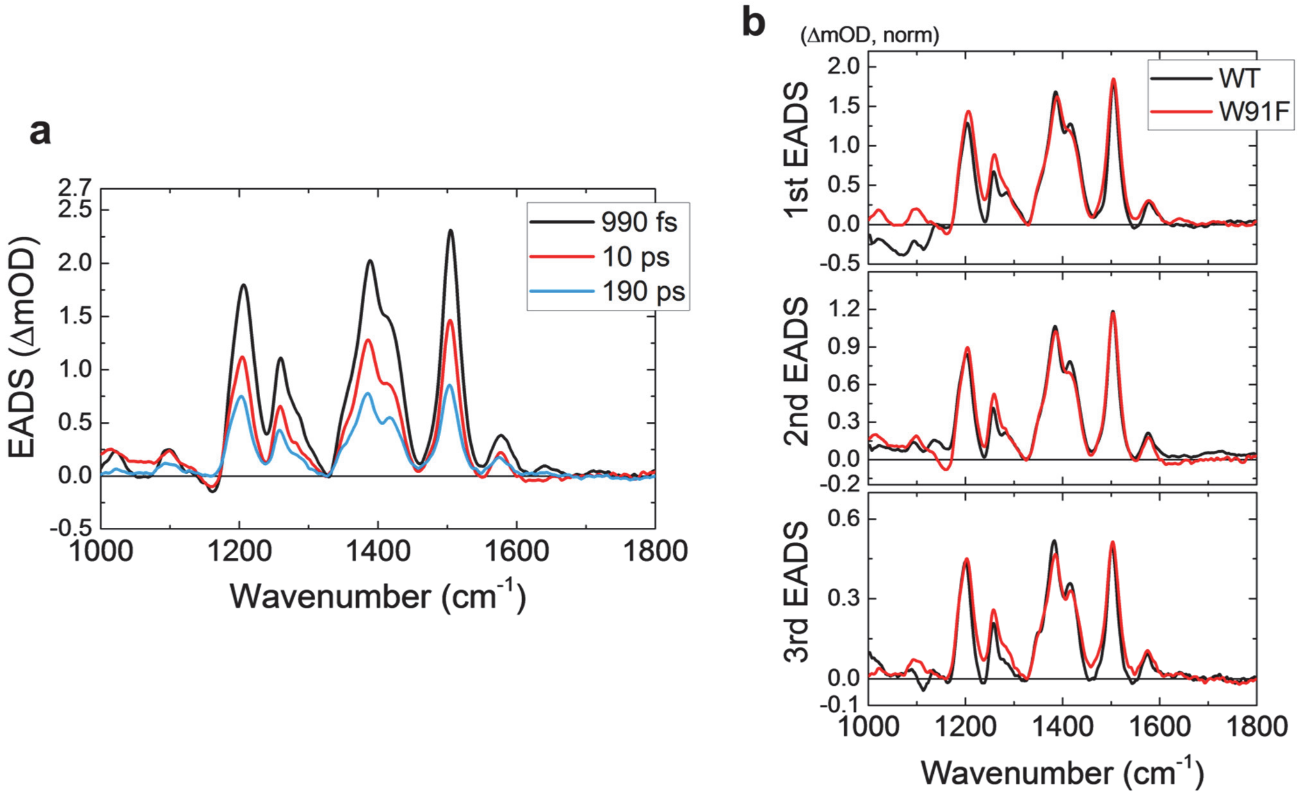

Figure S2. Comparison of FSRS of the DA state between WT Slr1694 BLUF photoreceptor and its W91F mutant. (**a**) EADS of the DA FSRS of the W91F mutant. (**b**) Comparison of normalized EADS of WT (black lines) and the W91F mutant (red lines) proteins. 1st (top), 2nd (middle) and 3rd (bottom) EADS are compared.

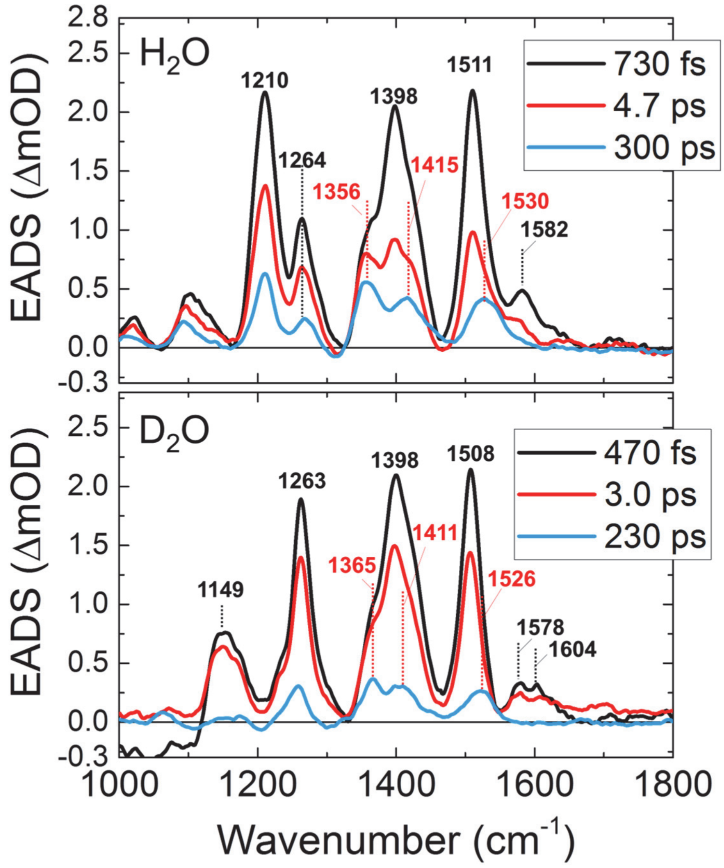

Figure S3. EADS of FSRS of the LA state on the W91F mutant in H_2O (top) and D_2O (bottom). The top figure is the same as the top panel of **Fig. 3b**. The differences in lifetimes between the samples in H_2O and D_2O are not considered to be significant.

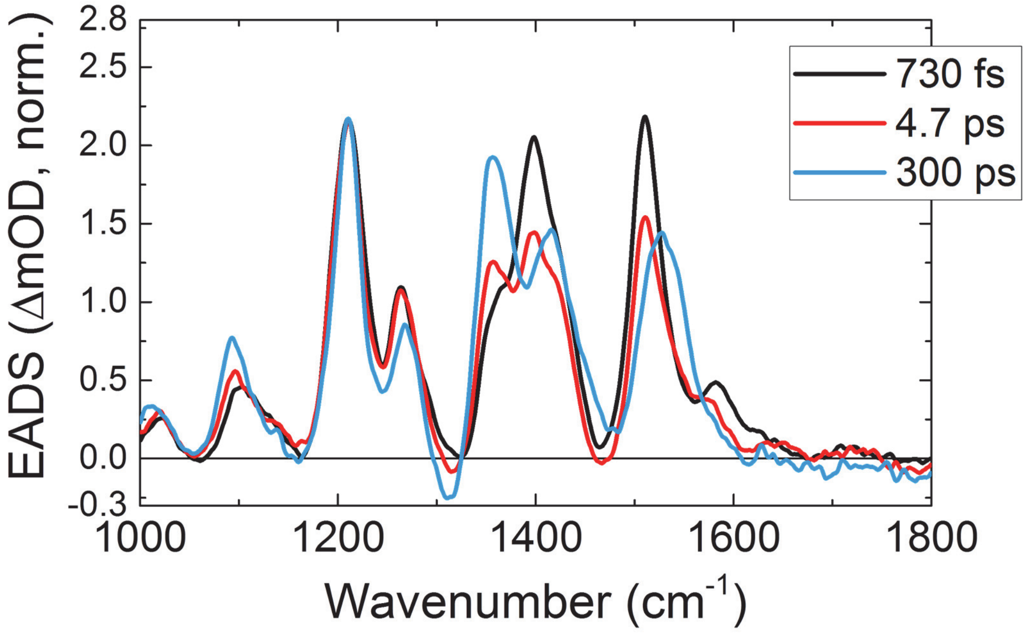

Figure S4. Normalized EADS of FSRS of the LA state on the W91F mutant in H_2O.

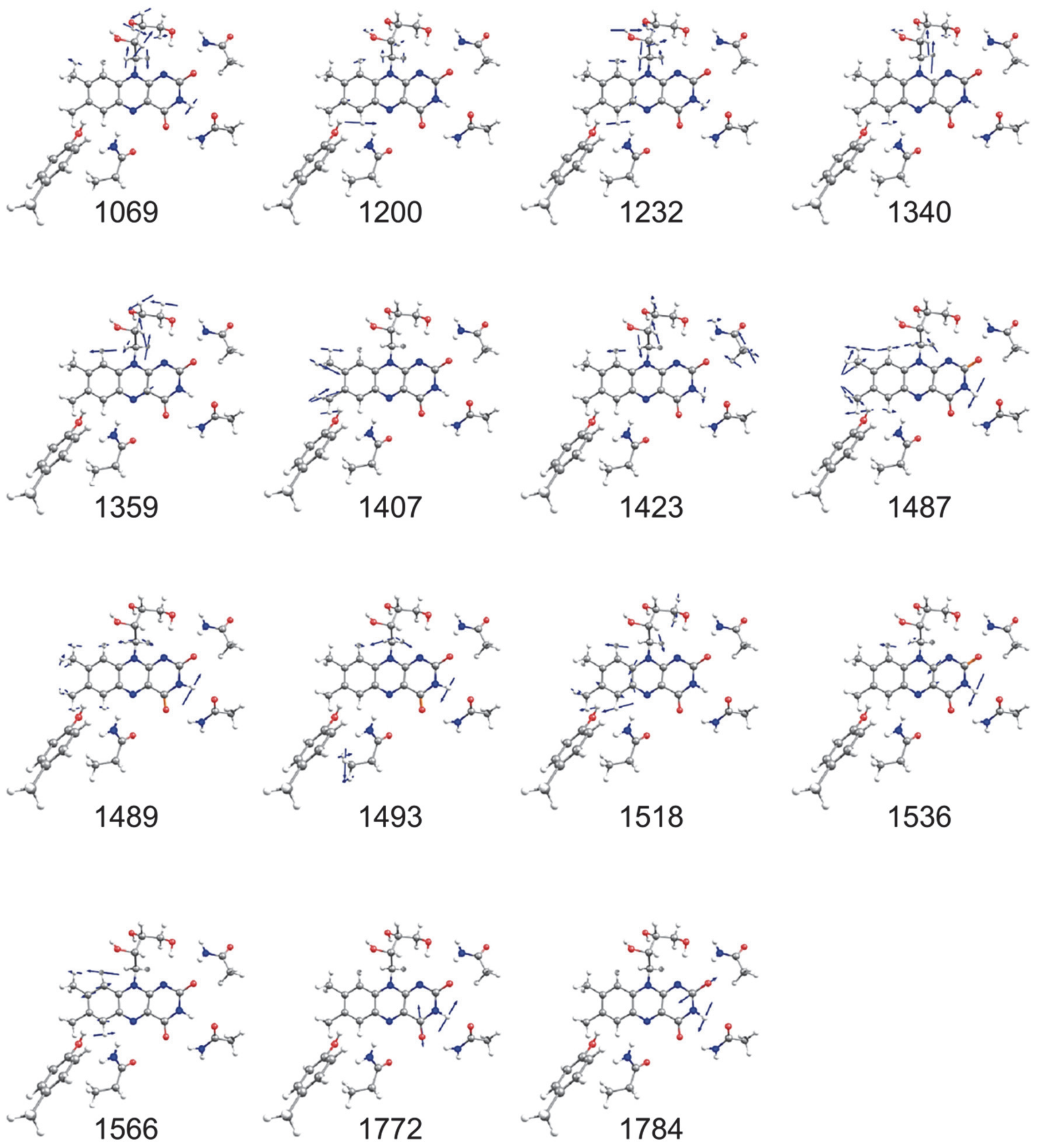

Figure S5. Selected vibrational modes of computational model on Model 1 FAD configuration.

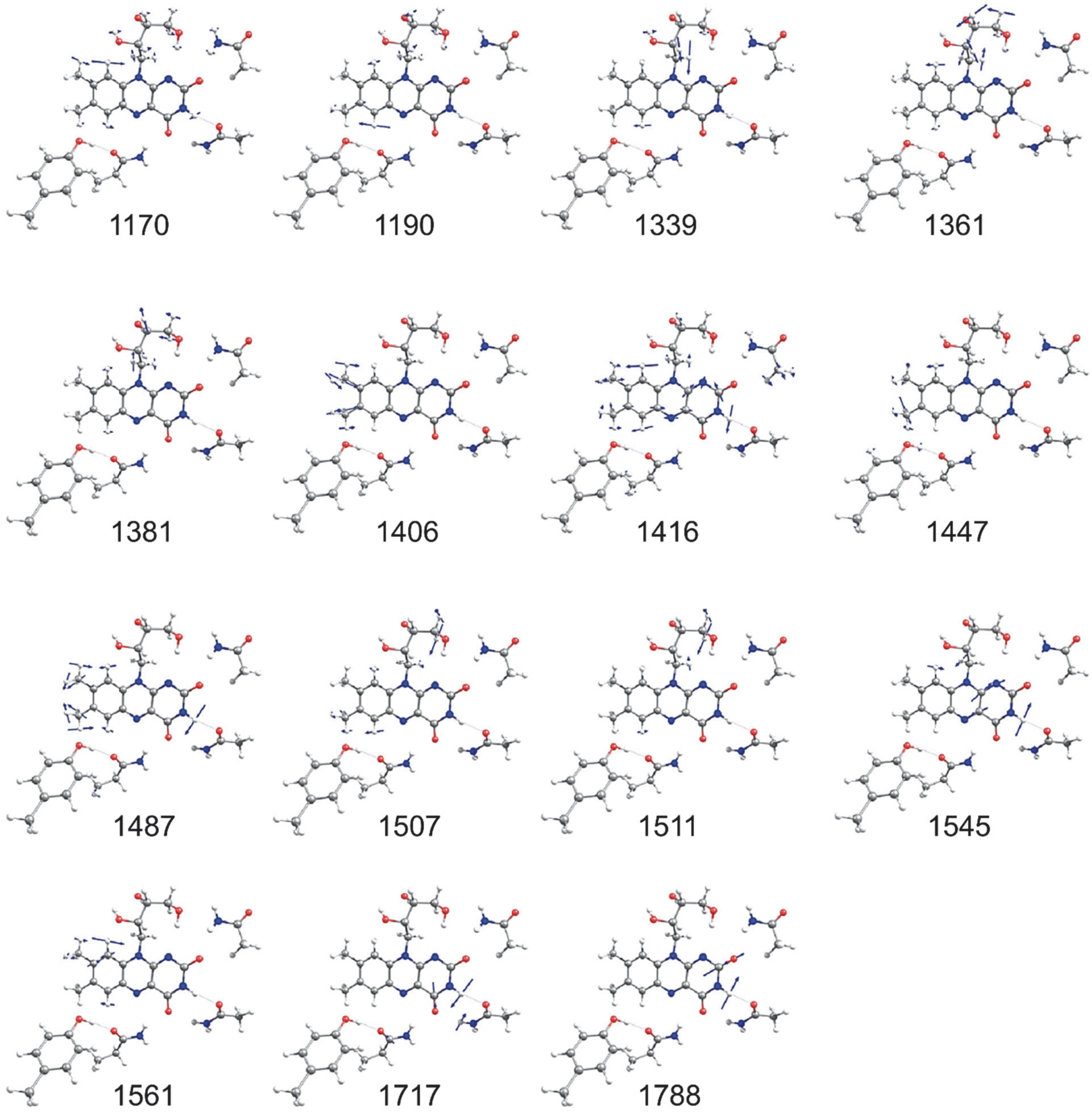

Figure S6. Selected vibrational modes of computational model on Model 2 FAD configuration.

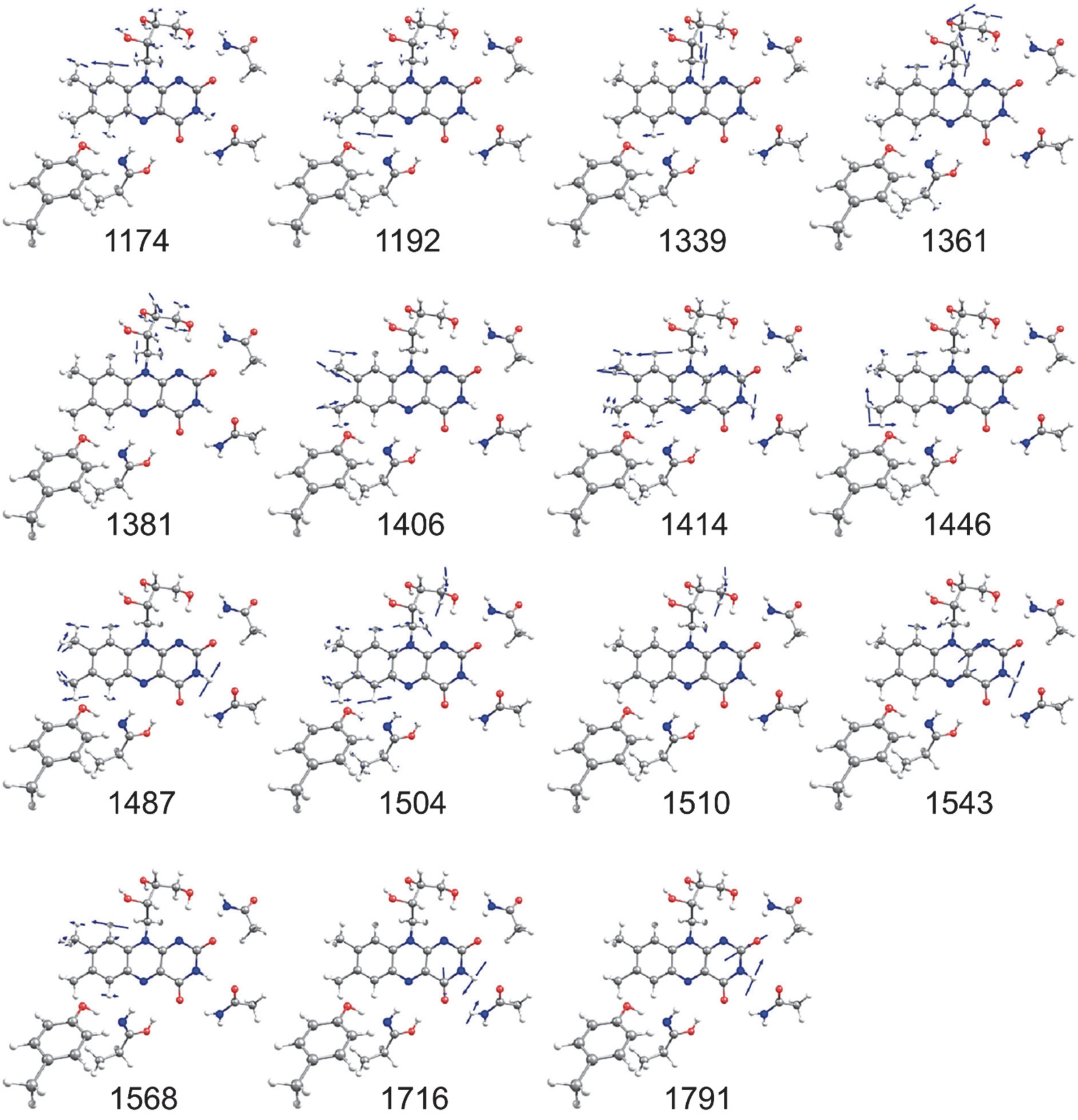

Figure S7. Selected vibrational modes of computational model on Model 3 FAD configuration.

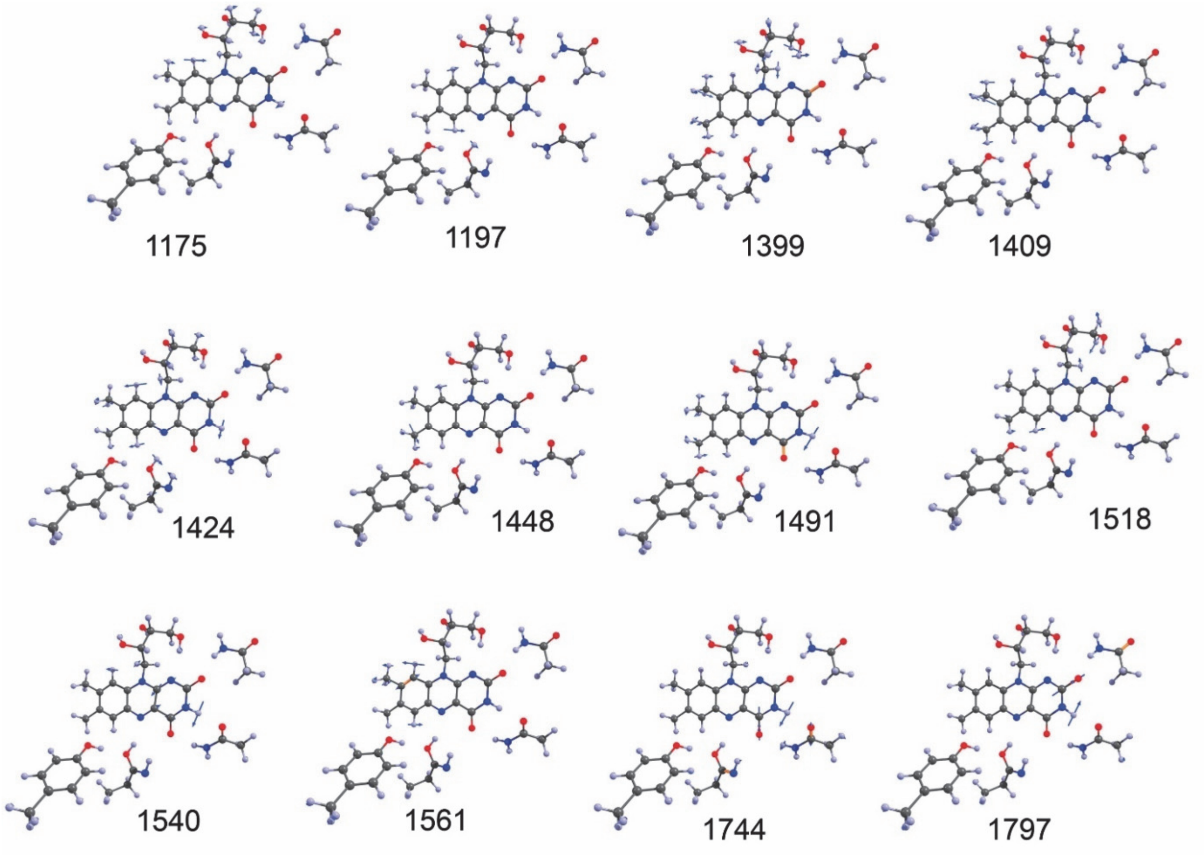

Figure S8. Selected vibrational modes of computational model on Model 4 FAD configuration.

Table S1. Frequency and Raman activity of computational model on Model 1.

Model 1			
H_2O		D_2O	
Freq., cm^{-1}	Raman Act., Å^4/AMU	Freq., cm^{-1}	Raman Act., Å^4/AMU
		1056	193
**1069**	**124**		
1089	41		
		1093	13
1104	16		
		1111	10
1114	5		
		1115	19
1125	3		
		1130	41
		1144	13
1146	33		
		1158	6
1169	91		
		1175	6
1183	8		
		1181	14
		1187	25
		1187	15
**1200**	**537**		
0		1205	849
**1232**	**539**		
1245	78	1245	375
1247	19		
1263	94		
1277	77	1275	80
		1285	12
		1290	20
1292	11		
		1308	67
1314	24		
		1321	71
		1328	102
**1340**	**119**		
		1347	111

Model 1			
H_2O		D_2O	
Freq., cm^{-1}	Raman Act., Å^4/AMU	Freq., cm^{-1}	Raman Act., Å^4/AMU
**1359**	**497**	1359	460
1367	92	1367	4
1382	18	1383	75
1387	10		
1396	17		
1397	6	1397	11
**1407**	**119**	1407	125
		1417	4
**1423**	**113**	1423	80
1426	36	1427	78
		1428	53
1430	2		
1439	8		
1450	62	1449	45
		1450	46
1453	24	1453	26
1454	24	1455	29
1456	22		
1472	27	1472	56
1473	76	1473	53
**1487**	**151**	1487	57
**1489**	**107**		
		1491	22
**1493**	**140**		
1510	54	1509	43
**1518**	**881**	1518	774
		1528	1087
**1536**	**675**		
**1566**	**1520**	1566	1510
		1719	69
		1753	185
**1772**	**190**		
		1778	407
**1784**	**404**		

Table S2. Frequency and Raman activity of computational model on Model 2.

Model 2			
H_2O		D_2O	
Freq., cm^{-1}	Raman Act., Å^4/AMU	Freq., cm^{-1}	Raman Act., Å^4/AMU
		1133	297
		1144	370
		1158	6
**1170**	**1191**		
		1175	157
		1181	205
1183	121		
		1187	366
**1190**	**2032**		
		1192	2249
1236	136		
1241	111		
1247	38		
		1250	85
1266	33		
		1270	264
1276	243		
		1286	35
		1291	7
1292	2		
		1307	106
1316	47		
		1320	67
		1335	128
**1339**	**720**		
		1346	796
**1361**	**710**	1360	780
1368	134	1368	3
**1381**	**366**	1382	461
1387	152		
1395	208		
1396	253	1396	81

Model 2			
H_2O		D_2O	
Freq., cm^{-1}	Raman Act., Å^4/AMU	Freq., cm^{-1}	Raman Act., Å^4/AMU
**1406**	**398**	1406	435
**1416**	**1285**	1416	395
		1417	597
		1419	739
1425	67	1424	218
		1428	56
1430	2		
1438	111		
**1447**	**831**	1447	890
1452	145	1452	115
		1456	193
1457	132		
1470	16	1470	17
1473	134	1472	118
1485	153		
**1487**	**1066**	1486	468
1493	42		
		1495	44
**1507**	**1727**	1506	1859
**1511**	**900**	1510	821
		1540	3421
**1545**	**2868**		
**1561**	**2235**	1561	2148
		1696	350
**1717**	**439**		
		1778	1025
**1788**	**835**		

Table S3. Frequency and Raman activity of computational model on Model 3.

Model 3			
H_2O		D_2O	
Freq., cm^{-1}	Raman Act., $Å^4/AMU$	Freq., cm^{-1}	Raman Act., $Å^4/AMU$
		1133	225
		1145	357
		1159	9
**1174**	**1139**	1175	141
		1182	85
1183	19		
		1188	434
**1192**	**2129**		
		1194	2289
1237	99		
1243	99		
1248	45		
		1251	51
1267	34		
		1272	277
1279	261		
		1286	45
		1291	12
1292	2		
		1308	108
1316	51		
		1320	80
		1336	113
**1339**	**824**		
		1346	958
**1361**	**835**	1360	897
1368	140	1368	5
**1381**	**478**	1382	592
1386	222		
1395	327		
1396	215	1396	64

Model 3			
H_2O		D_2O	
Freq., cm^{-1}	Raman Act., $Å^4/AMU$	Freq., cm^{-1}	Raman Act., $Å^4/AMU$
**1406**	**632**	1406	752
**1414**	**1063**	1414	1399
		1416	202
1425	57	1424	137
		1428	52
1430	6		
1438	114		
**1446**	**1008**	1446	987
1451	260	1451	250
		1454	210
1455	83	1455	11
1468	94	1468	102
1470	48	1470	29
1485	43		
**1487**	**1467**	1486	818
		1491	47
1492	187		
**1504**	**1968**	1504	2210
**1510**	**347**	1510	307
		1535	265
		1538	4266
**1543**	**3750**		
**1568**	**1604**	1568	1569
		1695	327
**1716**	**401**		
		1780	1092
**1791**	**896**		

Table S4. Frequency and Raman activity of computational model on Model 4.

Model 4			
H_2O		D_2O	
Freq., cm^{-1}	Raman Act., Å^4/AMU	Freq., cm^{-1}	Raman Act., Å^4/AMU
		1147	544
1149	7		
1152	16		
		1161	30
**1175**	**1552**		
		1177	9
		1196	262
**1197**	**1652**		
		1203	2591
**1237**	**315**		
		1249	31
1260	259		
1264	26		
		1280	312
1282	60		
1285	289		
		1291	44
		1299	100
1301	21		
		1308	166
1316	84		
		1321	48
		1338	173
1341	371		
1344	78		
		1347	704
**1359**	**838**		
		1361	510
		1367	7
1369	42		
1379	184		
		1383	289
1389	187		
1397	17		
		1398	54
1399	232		
1409	306	1409	319
		1419	174

Model 4			
H_2O		D_2O	
Freq., cm^{-1}	Raman Act., Å^4/AMU	Freq., cm^{-1}	Raman Act., Å^4/AMU
**1424**	**793**		
		1426	422
		1427	135
1428	131	1428	272
		1431	590
1432	28		
1442	117		
**1448**	**951**	1448	1013
		1454	181
1454	228		
		1459	218
1461	129		
1471	21	1471	20
1473	31	1473	31
		1478	135
1479	70		
1479	122		
1489	85		
		1490	680
**1491**	**1579**		
		1496	183
1497	33		
		1513	589
**1514**	**601**		
		1517	971
**1518**	**1570**		
		1533	1069
		1535	3694
**1540**	**3352**		
		1560	2681
**1561**	**2813**		
		1695	191
1718	172		
		1729	116
1744	231		
		1767	171
		1786	901
**1797**	**773**		

Table S5

Primer	Sequence 5’-3’	Manufacturer
DgltB-fwd	TAACCGATGC GAAAAGGACA ACAAGGGGGC GAATGCGAGG CGCGCGTATG gTgTAggCTg gAgCTgCTTC	Sigma
DgltB-rev	TAAACATTCTGACTCATTGTTGCTACCCCTTACTGCGCCT GCACGCGCAA CggCTgACAT gggAATTAgC	Sigma
DybaS-rev	ACCGTTATTA CCTTCCGTGT TCATCATGAT CAGCCCTTAA ACACGTTATA CggCTgACAT gggAATTAgC	Sigma
DybaS-fwd	GGGGTGAGGA ATTACCTCCC GCATCTATAA AAAGGAGTTA ACAAAAGATG gTgTAggCTg gAgCTgCTTC	Sigma
DyneH-rev	CTGATATACT CGCAGGTCTT TTCAGACCTG CGGTCCAGGA GTAGAAAGTG CggCTgACAT gggAATTAgC	Sigma
DyneH-fwd	ACGCGCGAAG AGTGGATCGA GAGACTGCAT TAATAAACCG AACGCCCTAA gTgTAggCTg gAgCTgCTTC	Sigma
HgltB-rev	GGAAAACGGCTCGTAAATTTC	Invitrogen
HgltB-fwd	GCTTGCCATTTGACCTGTATC	Invitrogen
HyneH-rev	GGAACTGGCTAATGATAGTG	Invitrogen
HyneH-fwd	CGGAATGTTATGCCACTTAG	Invitrogen
HybaS-rev	CATAACCGGAAAACATCGCC	Invitrogen
HybaS-fwd	GTTAAACCTTCCAGCAAGGG	Invitrogen

References

1. Weigel, A.; Dobryakov, A.; Klaumunzer, B.; Sajadi, M.; Saalfrank, P.; Ernsting, N. P., Femtosecond stimulated Raman spectroscopy of flavin after optical excitation. *J Phys Chem B* **2011,** *115* (13), 3656-80.
2. Gauden, M.; van Stokkum, I. H.; Key, J. M.; Luhrs, D.; van Grondelle, R.; Hegemann, P.; Kennis, J. T., Hydrogen-bond switching through a radical pair mechanism in a flavin-binding photoreceptor. *Proc Natl Acad Sci U S A* **2006,** *103* (29), 10895-900.
3. Mathes, T.; Zhu, J.; van Stokkum, I. H. M.; Groot, M. L.; Hegemann, P.; Kennis, J. T. M., Hydrogen Bond Switching among Flavin and Amino Acids Determines the Nature of Proton-Coupled Electron Transfer in BLUF Photoreceptors. *The Journal of Physical Chemistry Letters* **2012,** *3* (2), 203-208.
